# Intracellular IL-24 ameliorates lipid metabolic disorders in metabolic dysfunction-associated steatohepatitis by restoring the autophagy-lysosome pathway

**DOI:** 10.1007/s00018-025-05940-1

**Published:** 2025-11-25

**Authors:** Jiawei Cui, Zhandong Lin, Mengjiao Sun, Yuyuan He, Yaoyao Mao, Congyue Zhang, Yue Shi, Yukai Chen, Shaoya Li, Ying Zhang, Qianqian Zheng, Yuemin Nan

**Affiliations:** 1 Department of Traditional and Western Medical Hepatology, Hebei Provincial Key Laboratory of liver fibrosis in chronic liver diseases, Hebei Medical University Third Hospital, Shijiazhuang, 050051 Hebei P.R. China; 2 Hebei International Joint Research Center for Liver Cancer Molecular Diagnosis, Hebei International Science and Technology Cooperation Base, Shijiazhuang, 050051 Hebei P.R. China

**Keywords:** Metabolic dysfunction-associated steatohepatitis, IL-24, Autophagy-lysosome, Metabolism, AMPK/mTOR/TFEB axis

## Abstract

**Background:**

Metabolic dysfunction-associated steatohepatitis (MASH) is associated with impaired hepatic autophagy, but its key regulators remain unclear. This study delineates IL-24 as a regulator of autophagy in MASH.

**Methods:**

IL-24 was identified via bioinformatics in patient datasets and validated in metabolic dysfunction-associated steatotic liver disease (MASLD) patient sera. A high-fat high-fructose diet (HFFD) induced MASH in mice, with IL-24 overexpression via adeno-associated virus. Functional assessments were performed both in vivo and in primary hepatocytes using immunohistochemistry, Western blot, immunofluorescence, dual-fluorescence autophagic flux tracking, and multi-omics analyses.

**Results:**

MASLD patients and animal models showed significantly lower IL-24 expression, with levels inversely related to disease severity. IL-24 intervention improved liver steatosis, inflammation, fibrosis, and insulin resistance in MASH mice. Mechanistically, IL-24 mediates hepatocyte autophagy and alleviates lipid accumulation through the IL-22R1/IL-20R2 receptor complex. It activated AMP-activated protein kinase (AMPK), suppressed mechanistic target of rapamycin (mTOR), enhanced transcription factor EB (TFEB) nuclear translocation (as evidenced by reduced microtubule-associated protein 1 A/1B-light chain 3-II/sequestosome 1), restored autophagy-lysosome function, and increased lipid degradation. Multi-omics analysis indicated increased fatty acid oxidation and decreased glucose metabolism. Notably, KEGG enrichment analysis revealed significant association of differential metabolites with autophagy-related pathways, corroborating findings from transcriptomics and the AMPK/mTOR/TFEB regulatory axis.

**Conclusion:**

IL-24 preferentially utilizes the IL-22R1/IL-20R2 receptor complex to modulate the AMPK/mTOR/TFEB axis, thereby inducing autophagic-lysosomal activation, regulating glucose and lipid metabolism, and ameliorating hepatic lipid accumulation in MASH. These findings highlight IL-24 as a novel therapeutic target for MASH.

**Graphical Abstract:**

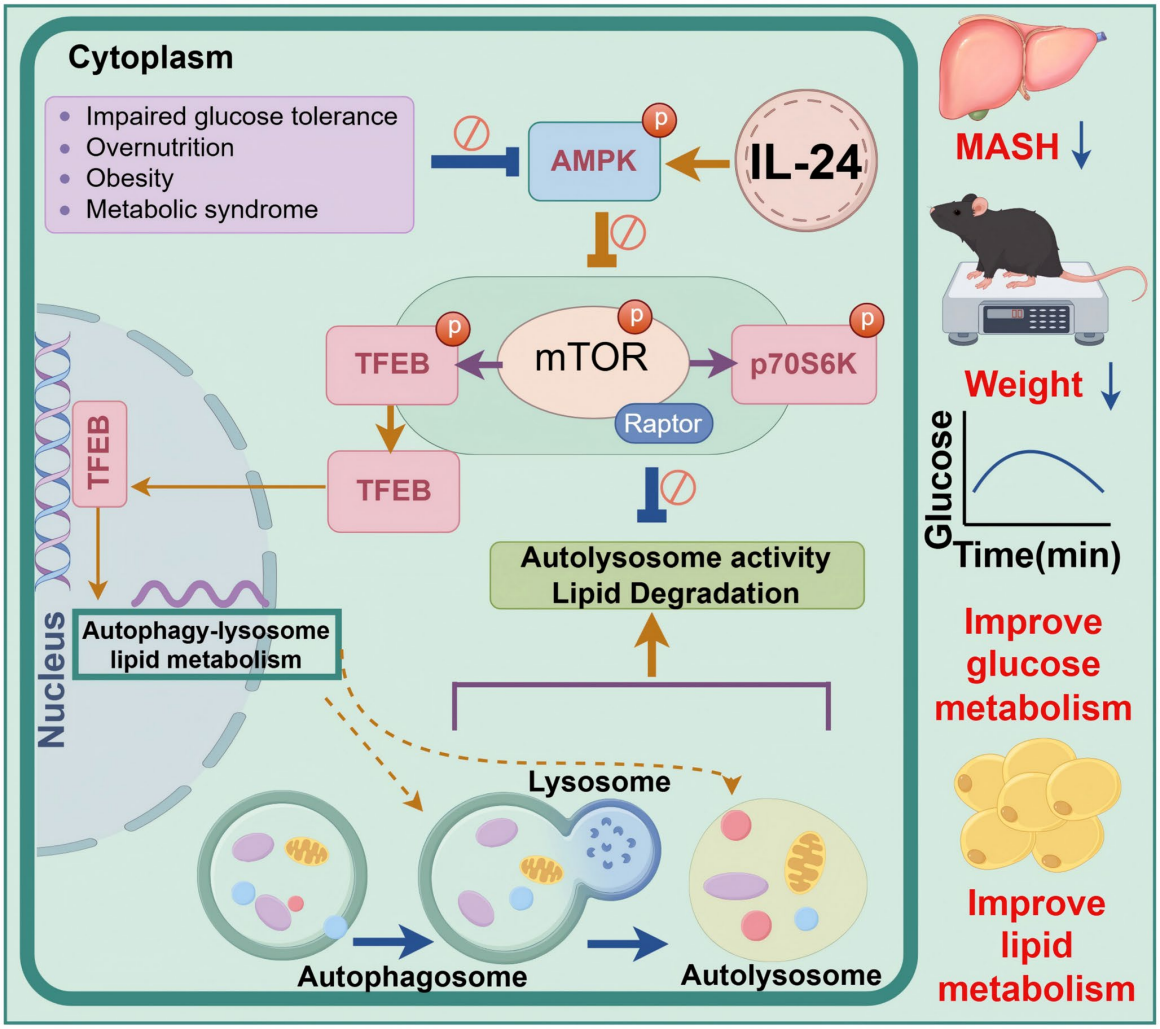

**Supplementary Information:**

The online version contains supplementary material available at 10.1007/s00018-025-05940-1.

## Introduction

Nonalcoholic Fatty Liver Disease (NAFLD), a condition long defined by hepatic lipid accumulation, is now recognized as a metabolic disorder with systemic implications. In response to this enhanced understanding, the international hepatology community has progressively refined its terminology since 2020. Following the adoption of metabolic-associated fatty liver disease (MAFLD), an international expert panel proposed a new nomenclature in June 2023 - metabolic dysfunction-associated steatotic liver disease (MASLD), with nonalcoholic steatohepatitis (NASH) updated to metabolic dysfunction-associated steatohepatitis (MASH) to better reflect its metabolic pathogenesis [1]. MASLD exhibits close bidirectional relationships with metabolic disorders including obesity, diabetes, and cardiovascular events [2]. In pathological progression, excessive accumulation of free fatty acids in hepatocytes not only induces lipotoxicity but also exacerbates liver injury through oxidative stress and inflammatory cascades, potentially progressing to hepatic fibrosis, cirrhosis, and even hepatocellular carcinoma [3].

Autophagy, a vital lysosome-mediated process, entails the enclosure of cytoplasmic contents within double-membrane autophagosomes marked by microtubule-associated protein light chain 3 (LC3) lipidation, followed by fusion with lysosomes to create autolysosomes where these contents are degraded by acidic proteases, hydrolases, and lipases – a process collectively known as the autophagy-lysosome pathway [4]. Growing evidence underscores the importance of autophagy in regulating lipid metabolism, with lipid droplets being specifically degraded through lipophagy [5]. Studies indicate that lipotoxic fatty acids, such as palmitate, disrupt autophagosome-lysosome fusion, thereby suppressing autophagic flux [6]. This impairment of autophagy is thought to exacerbate hepatic lipid accumulation, further promoting the progression of fatty liver disease. Transcription factor EB (TFEB) is a key transcriptional regulator of lysosome biogenesis and autophagy, and its activity is controlled by mammalian target of rapamycin (mTOR)-mediated phosphorylation [7]. Under conditions of nutrient abundance, elevated ATP levels suppress AMPK activity, thereby releasing its inhibition of mTOR, which subsequently phosphorylates TFEB, leading to cytoplasmic retention of TFEB and inhibition of autophagy. Conversely, during energy deprivation, accumulated AMP activates AMPK, which inhibits mTOR, resulting in TFEB dephosphorylation, nuclear translocation, and subsequent activation of lysosomal gene expression to augment autophagic flux [8]. Thus, the dysregulation of the AMPK/mTOR/TFEB axis likely constitutes a critical mechanism underlying autophagy impairment in MASLD.

Interleukin-24 (IL-24), a member of the IL-10 superfamily first identified as a negative regulator of human melanoma, has demonstrated bystander antitumor effects without cytotoxic activity toward normal cells [9–11]. IL-24 has been shown to downregulate several cardiovascular disease-associated genes, including angiotensinogen, endothelin-1, PDGF, and ATRAP, thus suggesting a role in cardiovascular protection [12, 13]. IL-24 is known to signal through two receptor complexes (IL20R1/IL20R2 and IL22R1/IL20R2), and exerts its effects in hepatocytes primarily through the IL22R1/IL20R2 rather than the IL20R1/IL20R2 complex [14]. Previous studies indicate that IL-24 can function as an autophagy inducer in various disease settings by suppressing the mTOR signaling pathway [15, 16], and it has exhibited hepatoprotective effects in experimental models of liver injury induced by thioacetamide, carbon tetrachloride, and acetaminophen [17, 18]. However, the specific impact of IL-24 on hepatic autophagic flux and lipid degradation in MASLD remains an open question.

Through bioinformatics screening of Gene Expression Omnibus (GEO) datasets, we identified a panel of dysregulated autophagy-related genes in MASH. Based on their statistical significance, poorly understood functions, and literature-supported potential roles, we selected the cytokine IL-24 as a candidate target for further investigation and explored its potential mechanisms in the pathogenesis of MASH. Our results demonstrate that IL-24 effectively alleviates MASH by enhancing autophagic flux and promoting lipid degradation through the AMPK/mTOR/TFEB signaling axis, which regulates the autophagy-lysosome pathway. These findings offer a novel diagnostic marker for MASH and position IL-24 as a promising therapeutic strategy for its prevention and treatment.

## Materials and methods

### Bioinformatics analysis

Gene expression data were obtained from three datasets (GSE48452 [19], GSE63067 [20], and GSE89632 [21]) in the GEO database [22], encompassing 154 liver tissue samples (82 NAFLD patients and 72 healthy controls) for autophagy-related gene (ARG) expression analysis. Gene symbols were matched to corresponding probes, and expression values for genes with multiple probes were averaged. Subsequent preprocessing involved log2 transformation, followed by batch effect removal using the “removeBatchEffect” function and data normalization via the “normalizeBetweenArrays” function, all within the R limma package. The integrity of the integrated datasets was confirmed by principal component analysis (PCA). A comprehensive set of 246 ARGs was compiled from the Human Autophagy Database (HADb) and a detailed literature review. Differential expression analysis of ARGs (DE-ARGs) between the NAFLD and control groups was performed using the limma package [23], with statistical significance set at |logFC| >0.2 and a Benjamini-Hochberg adjusted *P* < 0.05. Visualization of the results was achieved through heatmaps, volcano plots, and boxplots generated using the “heatmap” and “ggplot2” packages in R. To optimize feature selection, we utilized least absolute shrinkage and selection operator (LASSO) regression, a regularization technique that enhances predictive accuracy by selecting variables through penalized optimization. The “glmnet” R package [24] was employed for a 10-fold cross-validation procedure to tune the penalty parameter and identify the most critical features. For the analysis of public datasets, the term NAFLD is retained due to insufficient metabolic criteria in the original sample annotations.

### Animals

Male C57BL/6J mice (6–8 weeks old, weight range 18–20 g) were sourced from HFK Bioscience (Beijing, China) for the purposes of this study. The animals were housed in a specific pathogen-free facility under a controlled 12-h light/dark cycle with unrestricted access to standard laboratory diet and water. Following random assignment to experimental groups, the study concluded with euthanasia of all mice under isoflurane anesthesia, collection of blood via cardiac puncture, and subsequent harvesting of liver tissues for further investigation.

### Developing animal models for therapeutic intervention

Following a 7-day acclimation period during which all mice were fed standard chow, the experimental group was placed on a high-fat high-fructose diet (HFFD) for 18 weeks to induce MASLD. The HFFD consisted of 60% of calories from fat (XTHF60, Jiangsu Xietong Medicine Bioengineering Co., Ltd., Jiangsu, China) and 20% (w/v) fructose in the drinking water (D809612, Macklin, Shanghai, China). The control group was maintained on a normal chow diet (NCD) providing 10% of calories from fat (XTCON50J, same manufacturer) for the same duration [25].

The full-length IL-24 (NM_053095) cDNA was PCR-amplified and inserted into the pHBAAV-CMV-MCS-3flag AAV vector. The resulting IL-24 overexpression (oe) plasmid was then packaged into adeno-associated virus serotype 8 (AAV8) to generate the AAV8-IL-24 vector (AAV8-m-IL-24-3xflag-Null). Both the AAV8-IL-24 and the control AAV8-Null vectors were obtained from HanBio (Shanghai, China). At weeks 6 and 12 of the dietary intervention, mice were administered intravenously via the tail vein with 1 × 10^11^ viral genomes of AAV8-IL-24 or AAV8-Null in 100 µL of PBS.

### Clinical data collection

MASLD patients (*n* = 300) were recruited from the Department of Traditional and Western Medical Hepatology, Third Hospital of Hebei Medical University from January 2022 to December 2023. Inclusion criteria: (1) Diagnosis confirmed by abdominal ultrasound (independently assessed by two senior sonographers) and FibroScan (Controlled Attenuation Parameter, CAP >240 dB/m), consistent with the Guidelines for the prevention and treatment of metabolic dysfunction-associated (non-alcoholic) fatty liver disease (Version 2024) [26]; (2) Complete hepatic biochemical profiles available, with liver biopsy performed when necessary for histological assessment; (3) Signed informed consent. Exclusion criteria included: viral hepatitis (HBsAg/HCV antibody positivity), alcoholic liver disease, hepatic neoplasms, systemic inflammatory diseases, immunosuppressive therapy, and hepatic decompensation. 150 healthy controls were enrolled from routine health check-ups.

### Extended molecular biology methods

Methodological details (including primary mouse hepatocytes isolation and cell culture, construction and transfection of lentiviral vectors, cell transfection, construction of the cellular model, histopathological examination, qPCR, immunohistochemical staining, immunofluorescence staining, lysotracker red and bodipy 493/503 staining, autophagic flux analysis, flow cytometry, electron microscopy, western blot, transcriptomic analysis, metabolomics analysis, blood chemistry and cytokine quantification) have been archived in the Supplementary Methods.

### Statistical analysis

Quantitative data were presented as mean ± standard deviation (SD). unless stated otherwise, and subjected to statistical analysis using a two-tailed unpaired Student’s t test, one-way or two-way ANOVA with Bonferroni’s post hoc test, or Spearman’s rank correlation coefficient. All analyses were performed using GraphPad Prism version 9.0 software, and a p-value less than 0.05 (*P* < 0.05) was considered statistically significant.

## Results

### Identification and validation of IL-24 as an autophagy regulator in MASLD pathogenesis

**Fig. 1:**
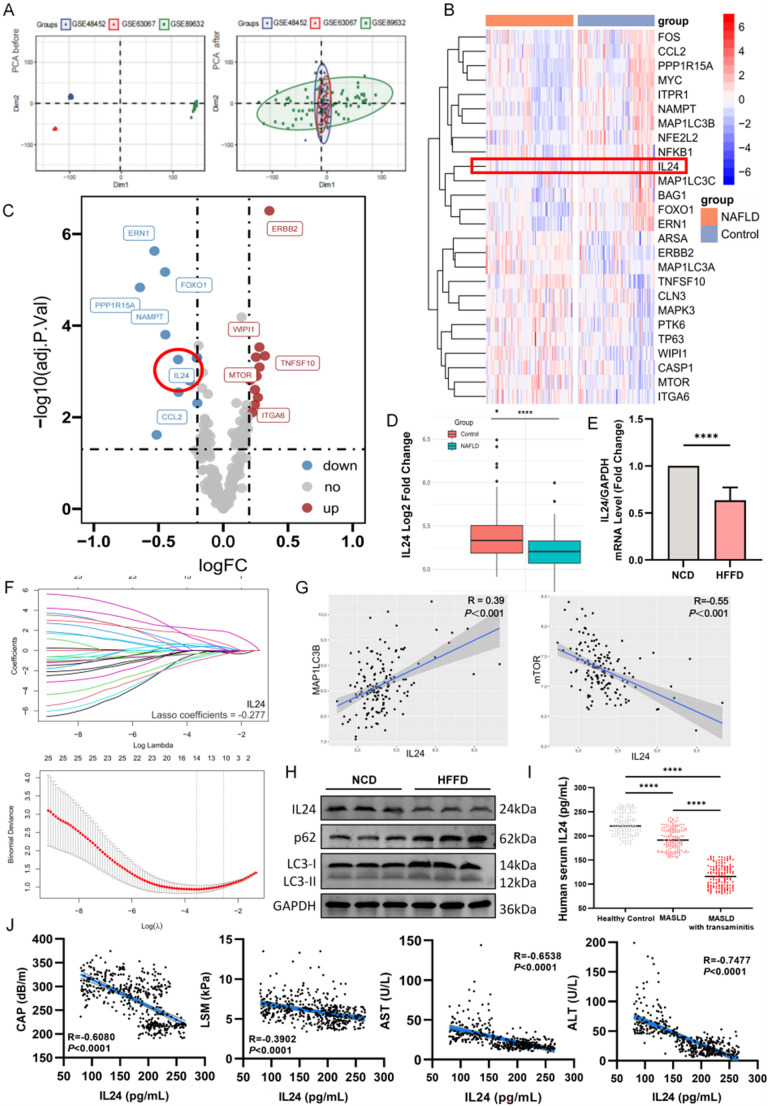
Functional characterization and identification of autophagy-related genes (ARGs) in metabolic dysfunction-associated steatotic liver disease (MASLD). (**A**) Principal component analysis (PCA) on hepatic steatosis datasets (GSE48452, GSE63067, GSE89632) demonstrates dimensionality reduction patterns pre- and post-normalization. (**B**, **C**) Differential expression of ARGs between healthy control (*n* = 72) and NAFLD (*n* = 82) liver samples is shown by a volcano plot and hierarchical clustering heatmap. (**D**) A boxplot using integrated GEO datasets (GSE48452, GSE63067, GSE89632) compares IL-24 expression in hepatic tissues of NAFLD patients and healthy subjects. (**E**) *IL-24* mRNA expression was confirmed by quantitative PCR (qPCR) in liver tissues of MASLD mice induced by a high-fat high-fructose diet (HFFD) versus controls on a normal chow diet (NCD) (*n* = 6/group). (**F**) The LASSO logistic regression algorithm was used to identify non-zero coefficient features significantly associated with NAFLD. (**G**) A scatter plot reveals significant correlations between IL-24 levels and the expression of key ARGs (*mTOR*, *MAP1LC3B*). (H) Western blotting shows significant changes in IL-24 and autophagy-related protein (LC3-II, p62) levels in liver tissues from HFFD mice compared to NCD controls (quantification in Fig. S1A, *n* = 6/group). (I) Measurement of human serum IL-24 levels in healthy controls, MASLD, and MASLD with transaminitis (*n* = 150/group). (**J**) Scatter plots reveal significant correlations between IL-24 levels and CAP (steatosis), LSM (fibrosis), AST, and ALT in MASLD patients. *P < 0.05; ****P < 0.0001

Based on transcriptomic data from human liver tissue samples in the GEO database, PCA illustrated tighter clustering of samples within each group following normalization (Fig. 1A), indicating effective batch effect correction and strong intra-group consistency, validating sample processing reliability. Differential expression analysis revealed 26 ARGs associated with NAFLD, including IL-24 (Fig. 1B-C). Hepatic *IL-24* mRNA levels were downregulated in NAFLD patients compared to controls (*P* < 0.0001; Fig. 1D), which was further supported by qPCR analysis in the HFFD-induced mouse model of NAFLD (Fig. 1E). LASSO regression analysis (Fig. 1F) selected “lambda.1se” as the optimal penalty parameter, with *IL-24* remaining a significant feature with a non-zero coefficient (β = −0.277), suggesting an inverse relationship with NAFLD progression. Correlation analyses (Fig. 1G) demonstrated a significant positive correlation between *IL-24* expression and the autophagy marker *MAP1LC3B* mRNA (*R* = 0.39, *P* < 0.001), and a significant negative correlation with *mTOR* expression (*R* = − 0.55, *P* < 0.001), implying a potential role for IL-24 in promoting autophagy through mTOR inhibition.

In the HFFD-induced mouse model of MASH, the observed bioinformatics data were consistent with in vivo findings: HFFD-fed mice showed reduced hepatic IL-24 expression along with increased protein levels of the autophagy receptors p62 and the autophagosome marker LC3-II (Fig. 1H; quantification in Fig. S1A), aligning with an inhibited autophagy-lysosome pathway [7]. Analysis of serum samples from MASLD patients recruited at our hospital revealed significantly lower serum IL-24 concentrations in MASLD patients (median [IQR]: 191.25 [171.97–212.61]) compared to healthy controls (220.545 [205.08–245.83]; *P* < 0.0001; Fig. 1I). Moreover, a further significant decrease in serum IL-24 (116.02 [95.48–135.75]) was observed in MASLD patients with concomitant transaminitis compared to those with MASLD alone (*P* < 0.0001). Correlation analysis (Fig. 1J) revealed that IL-24 expression levels demonstrated significant negative correlations with CAP values (*r* = −0.61, *P* < 0.0001), liver stiffness measurement (LSM, *r* = −0.39, *P* < 0.0001), AST (*r* = −0.65, *P* < 0.0001), and ALT (*r* = −0.75, *P* < 0.0001), indicating a negative association between IL-24 and hepatic steatosis severity, fibrosis progression, and liver injury in MASLD patients. Baseline clinical characteristics are detailed in Table S1.

### IL-24 improves metabolic dysfunction and hepatic pathology in murine MASH

**Fig. 2:**
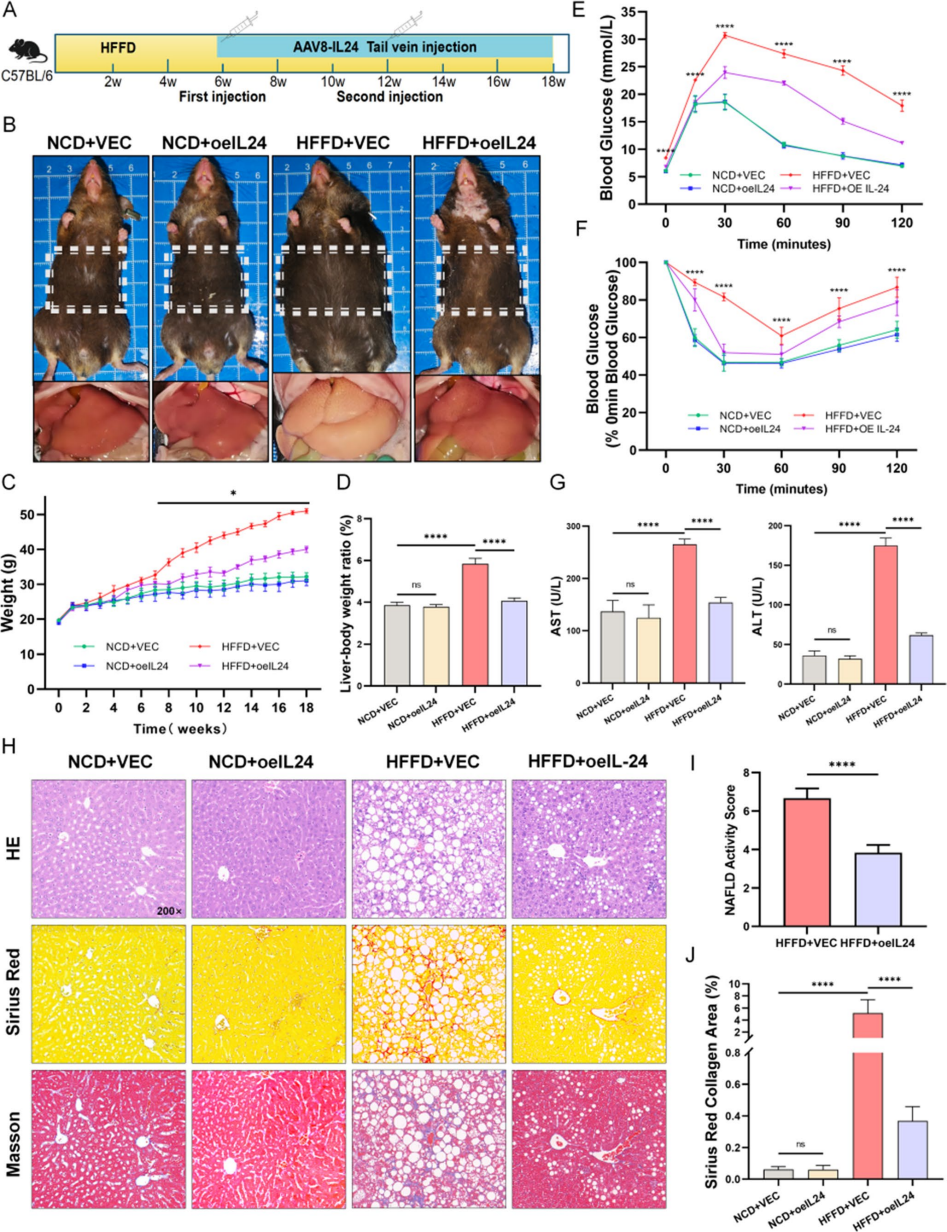
IL-24 overexpression reduces hepatic steatosis and liver injury in a HFFD mouse model. (**A**) Illustration of the experimental procedure and time course. Mice on NCD or HFFD for 18 weeks received intravenous AAV8-IL-24 or AAV8-Null vectors via the tail vein at 6 and 12 weeks. (**B**) Photographs depicting mouse body morphology and gross liver appearance after the 18-week treatment. (**C**) Body weight changes are plotted as a line chart over the 18-week experiment. (**D**) Bar charts show the percentage of liver-to-body weight across different groups (*n* = 6/group). Blood glucose monitoring during (**E**) intraperitoneal glucose tolerance tests (IPGTT, 2 g/kg glucose), and (**F**) insulin tolerance tests (ITT, 0.75 U/kg insulin) is presented in line charts (*n* = 6/group; quantification in Fig. S1B). (**G**) Bar charts show the serum ALT/AST levels (U/L) across different groups (*n* = 6/group). (**H**) Consecutive sections from the same liver lobe stained with H&E (steatosis, inflammation), Sirius red (collagen), and Manson’s trichrome (fibrosis) (*n* = 6/group). Scale bar: 200× magnification. (**I**) Quantification of the NAFLD activity score (NAS) and (**J**) the Sirius red-positive area (%). ns (not significant) *P* > 0.05; ****P < 0.0001

In the 18-week HFFD-induced C57BL/6 mouse model, IL-24 treatment was initiated at week 6 of the diet (Fig. 2A). Remarkably, IL-24-treated mice exhibited a significant divergence in body weight trajectory compared to the HFFD group from week 7 onward (Fig. 2C). By the conclusion of the study at week 18, IL-24 administration resulted in a significant reduction in both body weight and liver-to-body weight ratio relative to HFFD-fed mice (Fig. 2C, D). Intraperitoneal glucose tolerance test (IPGTT) and insulin tolerance test (ITT) results indicated that IL-24 effectively ameliorated HFFD-induced glucose intolerance and insulin resistance, as evidenced by statistically significant differences in the area under the curve (AUC) between the treatment groups (Fig. 2E-F; quantification in Fig. S1B).

Serum biochemical analysis revealed that IL-24 not only significantly reduced the elevated ALT and AST levels in HFFD-fed mice (Fig. 2G) but also concurrently downregulated the expression of pro-inflammatory cytokines TNF-α, IL-6, and IL-1β in serum (Fig. S1D). H&E staining of liver sections showed that HFFD feeding resulted in a NAS value of 6–7, which was significantly reduced by IL-24 intervention, as evidenced by decreased liver fatty degeneration, inflammatory infiltration, and hepatocyte ballooning (Fig. 2H, I). Further immunohistochemical staining demonstrated that IL-24 intervention markedly decreased positive signals of macrophage marker F4/80 and neutrophil marker Ly-6G in the liver, indicating suppressed infiltration of both macrophages and neutrophils (Fig. S1E). Additionally, Masson’s trichrome and Sirius red staining demonstrated that IL-24 treatment attenuated perisinusoidal fibrosis (Fig. 2J), which was supported by reduced protein levels of α-SMA and COL1α1 following IL-24 intervention (Fig. S1F).

### IL-24 alleviates hepatic lipid accumulation in MASH through AMPK/mTOR/TFEB-mediated autophagy-lysosome pathway activation

**Fig. 3:**
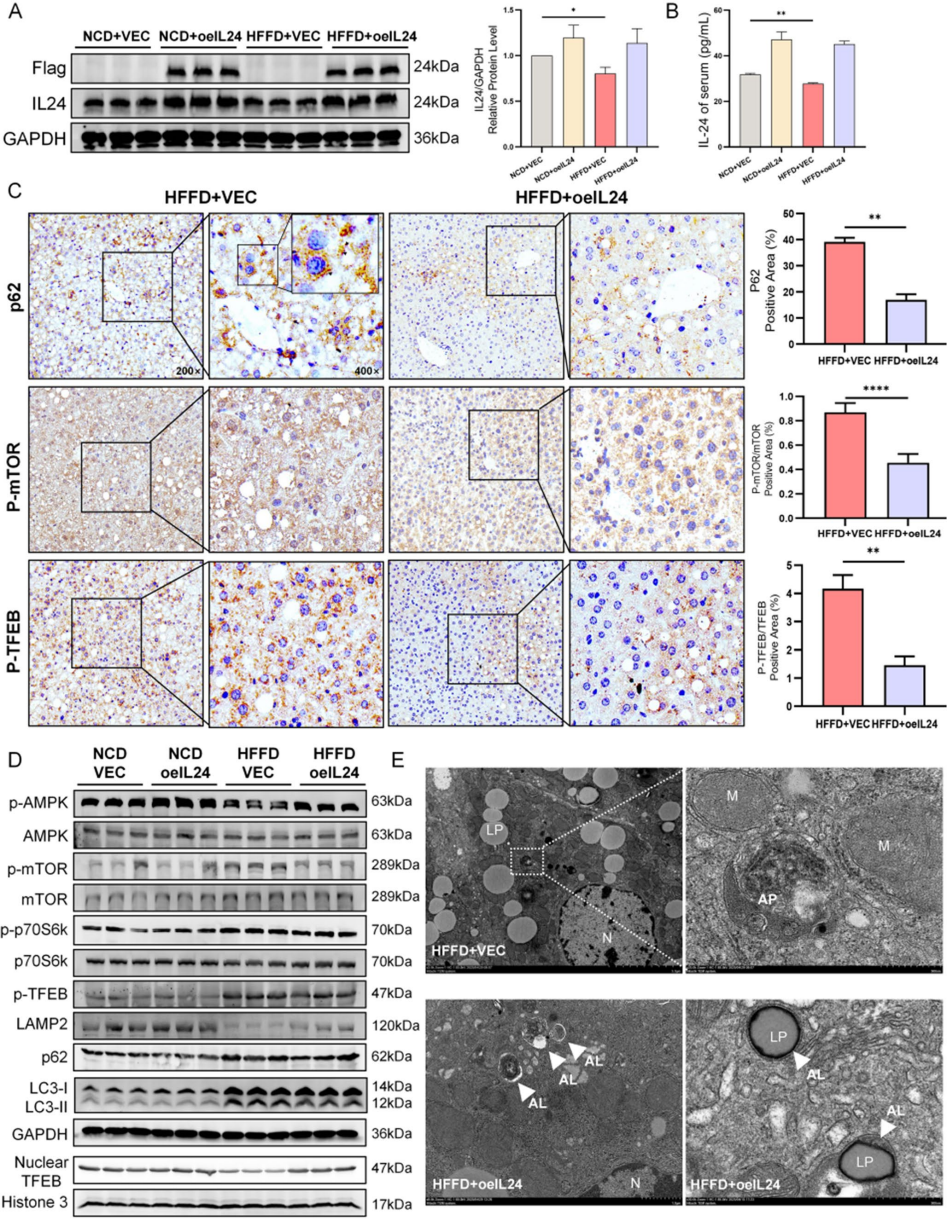
Hepatic IL-24 expression modulates the AMPK/mTOR/TFEB signaling network and corrects autophagic defects in HFFD-induced liver steatosis. (**A**) Expression levels of Flag-IL-24 and endogenous IL-24 determined by Western blot (*n* = 6/group). (**B**) Bar graph depicting the concentration of IL-24 (pg/mL) in the serum of experimental mice. (**C**) Immunohistochemical staining of p62, p-mTOR, and p-TFEB in representative liver tissues from the HFFD mouse model. IHC images and quantification of mTOR and TFEB are provided in Fig. S1G (*n* = 6/group). Scale bars are shown for 200× and 400× magnification. (**D**) Western blotting shows altered protein expression for the AMPK/mTOR/TFEB pathway and autophagy-related proteins (p-AMPK/AMPK, p-mTOR/mTOR, p-p70S6K/p70S6K, p-TFEB, LAMP2, p62, LC3-II and nuclear TFEB) in HFFD mouse livers. Quantification in Fig. S2A (*n* = 6/group). (**E**) TEM images showing hepatic autophagic vesicles. Key organelles are labeled: LD (lipid droplet), M (mitochondria), N (nucleus), AP (autophagosome), and AL (autolysosome). *P < 0.05; **P < 0.01

Confirmation of successful in vivo IL-24 overexpression was achieved by Western blot analysis of the Flag-tag, which also demonstrated significantly lower endogenous IL-24 expression in liver and serum of HFFD-fed mice compared to NCD controls (Fig. 3A-B). Immunohistochemical analysis (Fig. 3C) showed that IL-24 intervention significantly reduced p62, p-TFEB expression, p-mTOR/mTOR ratio, while increasing nuclear TFEB accumulation compared to the HFFD group, supporting the hypothesis that IL-24 enhances autophagy through modulation of mTOR-related pathways (IHC images and quantification of mTOR and TFEB are provided in Fig. S1G). Further Western blot analysis revealed that HFFD feeding resulted in increased expression of p-mTOR and p-TFEB, along with reduced p-AMPK and nuclear TFEB levels. These changes were accompanied by decreased LAMP2 expression and accumulation of LC3-II and p62, findings that are consistent with impaired autophagic-lysosomal function. IL-24 treatment alleviated these alterations, as evidenced by increased p-AMPK levels and decreased p-mTOR and p-TFEB expression, suggesting its potential role in regulating autophagy through the AMPK/mTOR/TFEB axis, a mechanism that was validated in our subsequent in vitro experiments. Furthermore, the simultaneous increase in LAMP2 and decrease in LC3-II and p62 are indicative of a potential restoration of autophagy-lysosome function (Fig. 3D; quantification in Fig. S2A).

TEM demonstrated substantial accumulation of lipid droplets and a scarcity of autophagosomes in hepatocytes from HFFD-fed mice. In contrast, liver sections from IL-24-treated mice showed a marked decrease in lipid droplets and an increase in autolysosomes, which contained lipid inclusions and were often surrounded by membranous structures, indicating the presence of lipophagy (Fig. 3E). These findings suggest a potential mechanism whereby IL-24 was found to exert protective effects against MASH progression by promoting autophagy, which was later demonstrated to reduce intrahepatic lipids through enhanced lipolysis and lipid oxidation.

### IL-24 enhances autophagic flux through AMPK/mTOR/TFEB pathway regulation

**Fig. 4:**
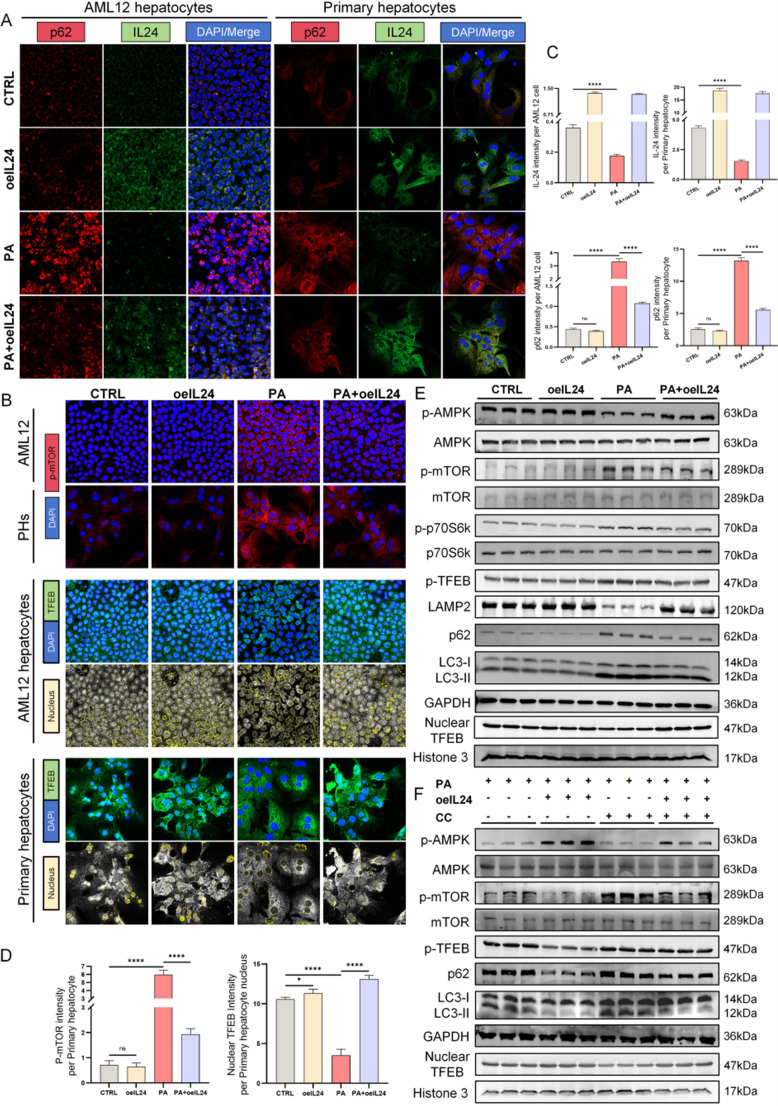
IL-24 enhances autophagy via the AMPK/mTOR/TFEB signaling axis in vitro. AML12 cells and mouse primary hepatocytes (PHs) were transduced with lentiviral vectors carrying IL-24 (LV-IL-24-PURO) or an empty vector control (LV-PURO) at an MOI of 20. Cells in the PA group were treated for 24 h with 500 µM PA conjugated to 20% fatty acid-free BSA, while control (CTRL) groups received an equivalent volume of 20% BSA vehicle. The AMPK inhibitor Compound C (CC, 1 µM) was treated alone or co-treated with PA for 24 h. Representative immunofluorescence staining for (**A**) p62 (red)/IL-24 (green) and (**B**) p-mTOR (red)/TFEB (green). DAPI (blue) was used for nuclear counterstaining. Scale bars: 200× magnification. (**C**) Quantification of p62 and IL-24 fluorescence intensity for both cell types. (**D**) Quantification of p-mTOR and nuclear TFEB signals in PHs (corresponding data for AML12 cells are provided in Fig. S2D) (*n* = 3/group). (**E**, **F**) Western blot analysis demonstrates altered expression of proteins in the AMPK/mTOR/TFEB pathway and autophagy markers (p-AMPK/AMPK, p-mTOR/mTOR, p-p70S6K/p70S6K, p-TFEB, LAMP2, p62, LC3-II and nuclear TFEB) in PHs. Quantification in Fig. S2F, Fig. S3A (*n* = 3/group). ns *P* > 0.05; ***P < 0.001; ****P < 0.0001

To corroborate the proposed mechanism of IL-24 at the cellular level, we performed experiments using AML12 and primary hepatocytes (PHs). Immunofluorescence analysis revealed a significant reduction in IL-24 expression upon palmitic acid (PA) stimulation in both AML12 cells and PHs compared to the control, whereas overexpression of IL-24 substantially reduced PA-induced expression of p62 and p-mTOR (Fig. 4A-D). Strikingly, IL-24-treated cells displayed significantly increased nuclear localization of TFEB (Fig. 4B, D; quantification in Fig. S2D for AML12 cells), suggesting enhanced nuclear translocation following TFEB dephosphorylation, potentially representing a cellular attempt to compensate for PA-mediated autophagy inhibition. Western blot analysis demonstrated that PA stimulation induced dose-dependent increases in LC3-II and p62 expression in PHs with escalating concentrations (Fig. S2E). Additionally, PA treatment led to reduced p-AMPK levels, enhanced p-mTOR and p-TFEB, diminished nuclear TFEB protein, and downregulated LAMP2 expression (Fig. 4E; quantification in Fig. S2F). These findings collectively indicate that PA treatment leads to molecular alterations consistent with impaired autophagic-lysosomal function, including the accumulation of autophagy substrates and inhibition of key regulatory kinases. Importantly, IL-24 intervention attenuated these PA-induced alterations.

To further dissect the signaling pathway, we evaluated the role of AMPK using compound C (CC), an AMPK inhibitor, in PHs. Compared to the PA group, cells exposed to both PA and CC exhibited a more pronounced inhibition of p-AMPK and a further increase in the expression of p-mTOR and p-TFEB, leading to reduced nuclear TFEB translocation and a more severe impairment of autophagic flux, evidenced by elevated LC3-II and p62 levels. Critically, IL-24 treatment was able to alleviate these effects even under PA + CC conditions (Fig. 4F; quantification in Fig. S3A; p-AMPK immunofluorescence in Fig. S3B). Collectively, these findings suggest that IL-24 may regulate autophagic-lysosomal function through the AMPK/mTOR/TFEB axis.

### Validation of IL-24-mediated autophagic flux restoration through the lysosomal pathway by multimodal analysis

**Fig. 5:**
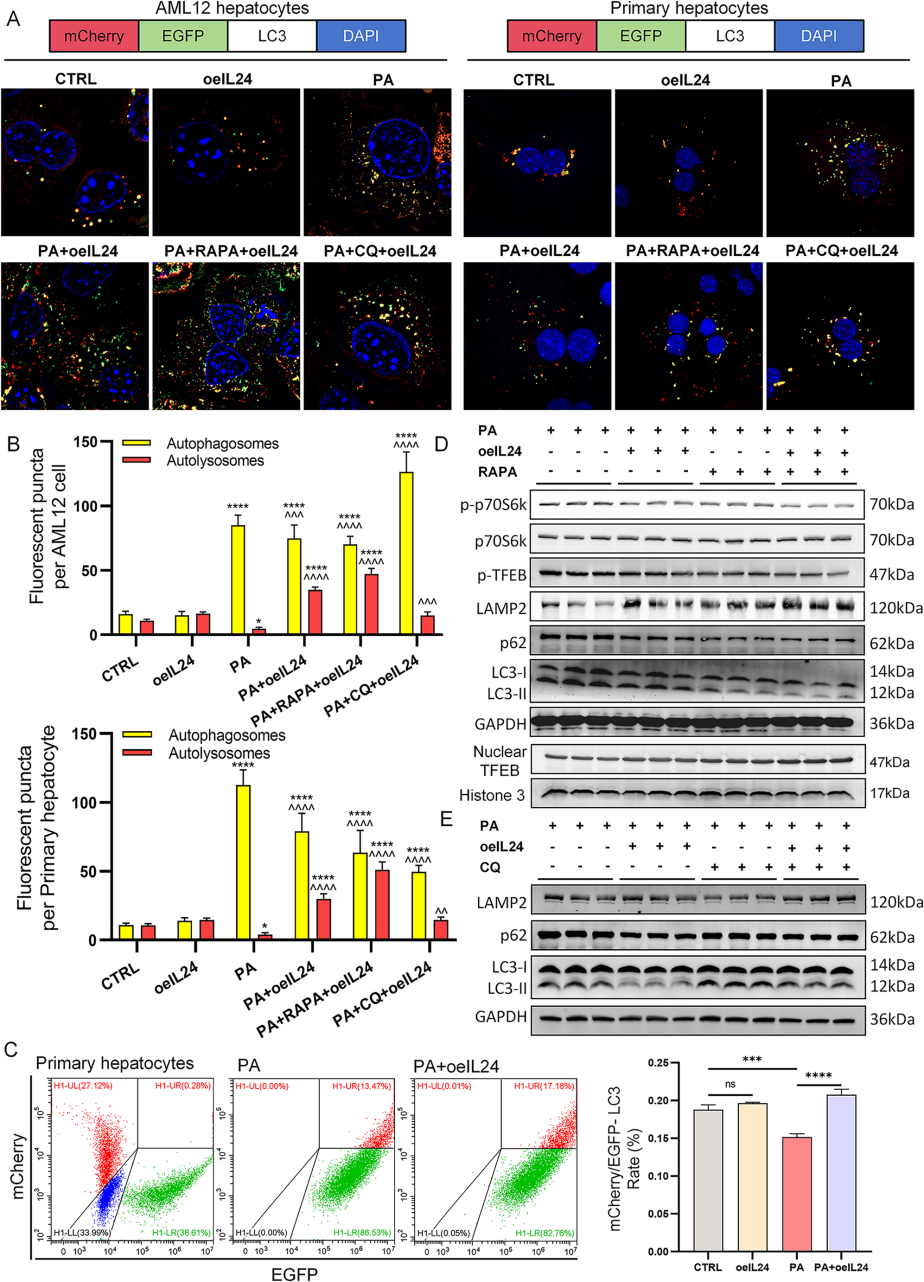
In vitro assessment of autophagic flux. AML12 cells and PHs were transduced with lentiviral vectors carrying IL-24 (LV-IL-24-PURO) or an empty vector control (LV-PURO) at an MOI of 20. Cells were exposed to 500 µM PA or vehicle (CTRL) for 24 h. Autophagy inhibitor CQ (10 µM) was applied alone or co-treated with PA for 24 h. Additionally, an autophagy agonist, rapamycin (RAPA, 0.25 µM), was used alone or co-treated with PA for 24 h. (**A**) Representative images of cells transduced with adenovirus (Ad-mCherry-EGFP-LC3; MOI 40, 24 h) display autophagosomes (yellow puncta, mCherry + EGFP) and autolysosomes (red puncta, mCherry). Scale bars: 630× magnification (oil immersion). (**B**) Quantification of puncta/cell (*n* = 10/group) is presented. (**C**) Flow cytometry analysis in PHs transduced with Ad-mCherry-EGFP-LC3 reveals the mCherry-to-EGFP fluorescence ratio (*n* = 3/group). (**D**, **E**) Western blotting indicates significant alterations in protein levels of the AMPK/mTOR/TFEB signaling network and autophagy-related proteins (p-p70S6K/p70S6K, p-TFEB, LAMP2, p62, and LC3-II) in PHs. Quantification in Fig. S3D, E (*n* = 3/group). *P < 0.05 vs. CTRL group; ^*P* < 0.05 vs. PA group; **/^^*P* < 0.01; ***/^^^*P* < 0.001; ****/^^^^*P* < 0.0001

To investigate the mechanism of IL-24 in regulating autophagic flux, we employed an mCherry-EGFP-LC3 dual-fluorescence reporter system in both AML12 and PHs. PA-stimulated cells exhibited a significant reduction in red puncta (autolysosomes) and an increase in yellow-green puncta (autophagosomes) compared to the control groups, indicating a blockage in autophagosome maturation. IL-24 intervention increased the intensity of the mCherry signal, suggesting a restoration of autolysosome formation. Co-administration of rapamycin (RAPA), an established autophagy inducer, further enhanced the red fluorescence, demonstrating a synergistic effect with IL-24 on autophagic flux. Following the addition of the autolysosome inhibitor chloroquine (CQ) to the PA + IL-24 treatment group, the number of autolysosomes decreased but remained significantly higher than in the PA-only group, as evidenced by a relative increase in red fluorescent puncta (Fig. 5A-B). Population-based quantification using flow cytometry (to avoid sampling bias from limited imaging fields [27]) confirmed that IL-24 overexpression significantly increased the percentage of mCherry-positive cells, further supporting the conclusion that IL-24 restores autophagic-lysosomal function (Fig. 5C, Fig. S3C shows control and oeIL-24 groups).

Consistent with the aforementioned results, Western blot analyses in PHs demonstrated that RAPA treatment following PA stimulation decreased p-p70S6K (indicating mTOR inhibition), reduced levels of p-TFEB, p62, and LC3-II, and increased LAMP2 expression and nuclear TFEB accumulation, collectively indicating restored autophagy-lysosome function. IL-24 intervention further enhanced these effects, indicating a potential synergistic action in promoting autophagosome-lysosome fusion. Remarkably, IL-24 significantly reduced p62 and LC3-II levels while increasing LAMP2 expression even under CQ treatment, providing functional evidence that IL-24 promotes autophagic flux by enhancing lysosomal pathway (Fig. 5D-E; quantification in Fig. S3D-E).

**Fig. 6:**
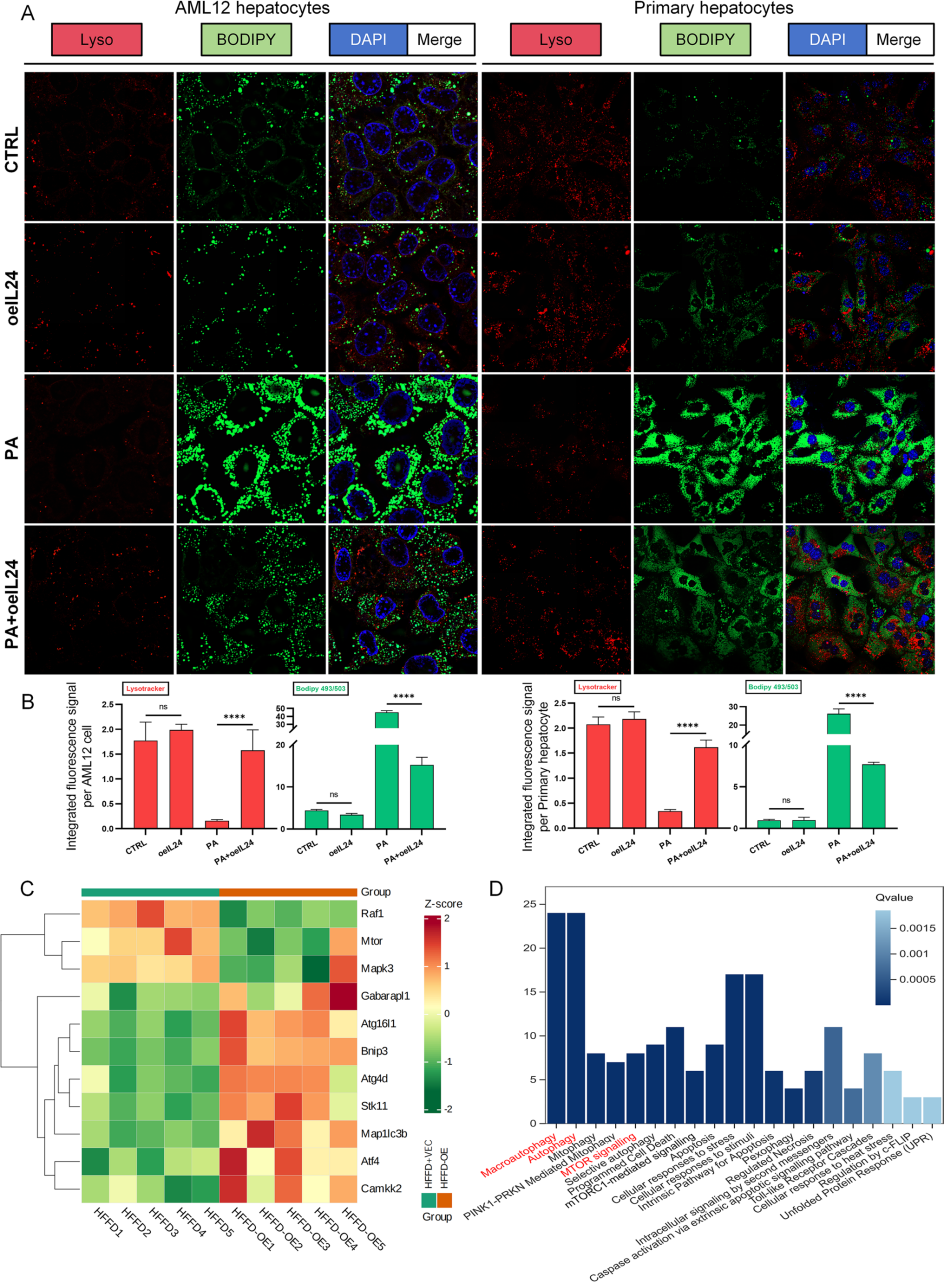
In vitro assessment of autophagy completion and in vivo bioinformatics analysis of ARGs. AML12 cells and PHs were transduced with lentiviral vectors carrying IL-24 (LV-IL-24-PURO) or an empty vector control (LV-PURO) at an MOI of 20. Cells were exposed to 500 µM PA or vehicle (CTRL) for 24 h. (**A**) Representative fluorescence images, utilizing dual labeling with LysoTracker (red) and BODIPY 493/503 (green), demonstrate the colocalization of acidic lysosomes and cellular lipids. (**B**) Bar graphs provide the quantified fluorescence intensity of LysoTracker (red) and BODIPY 493/503 (green) (*n* = 3/group). (**C**) Clustering heatmap reveals differential expression patterns of critical ARGs in vivo (*n* = 5/group). (**D**) Bar graph presents the Reactome enrichment results of ARGs in vivo (*n* = 5/group). ns *P* > 0.05; ****P < 0.0001

To assess the functionality of the late stages of the autophagy-lysosome pathway, we employed LysoTracker staining in both AML12 cells and PHs to visualize acidified autolysosomes and lysosomes. Upon co-staining with BODIPY 493/503, a marker for lipid droplets, PA-treated cells showed a significant decrease in LysoTracker red fluorescence, indicative of impaired lysosomal acidification. IL-24 intervention significantly restored the red fluorescence intensity while simultaneously reducing the abundance of BODIPY-labeled lipid droplets (Fig. 6A-B). Colocalization analysis further demonstrated a substantial reduction or complete disappearance of lipid droplet fluorescence in regions enriched with LysoTracker signal. This spatial pattern suggests a potential link between the restored lysosomal activity and the degradation of lipids. Collectively, these findings support the conclusion that the autophagy-lysosome pathway is a contributor to the IL-24-induced reduction of intracellular lipids, a finding further validated by multi-omics analyses.

###  Transcriptomic analysis reveals IL-24 regulates the core molecular network of autophagy in mice

Transcriptomic profiling of murine liver tissue provided further evidence for IL-24’s regulatory function in autophagy. Heatmap analysis revealed that IL-24 treatment led to the downregulation of genes associated with mTOR signaling and the upregulation of genes involved in autophagy (Fig. 6C). Reactome pathway enrichment analysis identified significant clustering of differentially expressed genes within key pathways, including “Macroautophagy” and “mTOR signaling” (Q-value < 0.05) (Fig. 6D). These expression patterns were consistent with earlier bioinformatic predictions derived from human samples (Fig. 1). Integrated analysis of transcriptomic data and prior experimental results supports a model in which IL-24 activates AMPK to suppress mTOR activity, promotes TFEB nuclear translocation (Figs. 3 and 4), thereby enhances autophagic flux while reducing lipid droplet accumulation.

### IL-24 mediates hepatocyte autophagy and alleviates lipid accumulation through the IL-22R1/IL-20R2 receptor complex

**Fig. 7:**
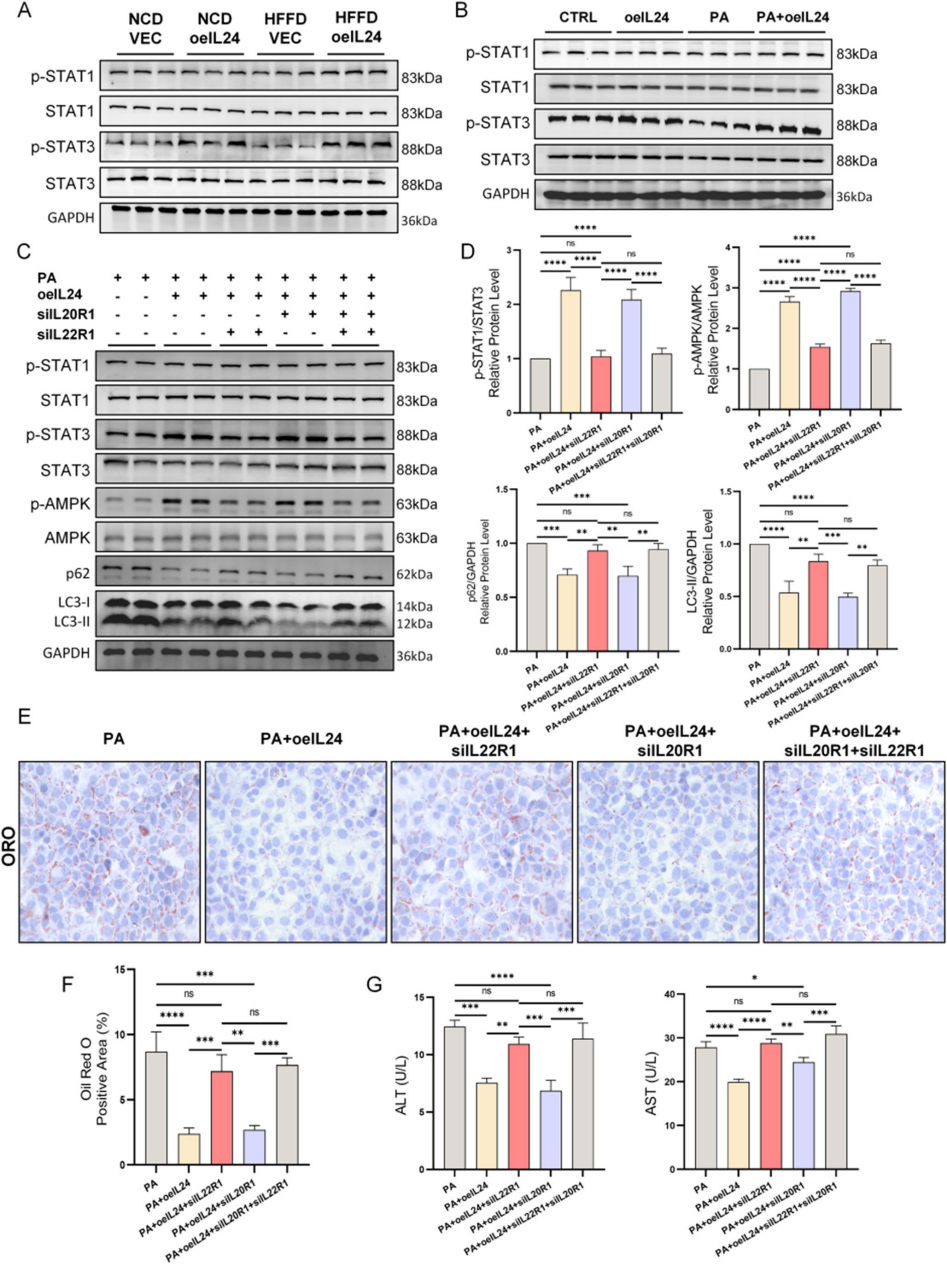
IL-24 mediates the hepatocyte autophagic-lysosomal pathway through the IL-22R1/IL-20R2 coreceptor complex rather than the IL-20R1/IL-20R2 complex. AML12 cells were initially transfected with lentiviral vectors carrying IL-24 (LV-IL-24-PURO) or an empty vector control (LV-PURO) at an MOI of 20. Following puromycin selection to establish stable lines, cells were subsequently transfected with siRNA to knock down IL20R1 and IL22R1 respectively. Western blot analysis detecting expression changes of key downstream molecules (p-STAT1/STAT1 and p-STAT3/STAT3) of IL-24 receptor complexes (IL20R1/IL20R2 and IL22R1/IL20R2) in vivo (**A**) (*n* = 6/group) and in vitro (**B**) (*n* = 3/group) models. Quantification in Fig. S4A, B. (**C**, **D**) Western blot analysis of IL-24 receptor downstream signaling molecules (p-STAT1, STAT1, p-STAT3, STAT3) and autophagy-related proteins (p-AMPK, AMPK, P62, LC3-II) in AML12 cells (*n* = 3/group). Quantification of p-STAT1/STAT1 ratio in Fig. S2C. Representative photomicrographs of Oil Red O-stained AML12 cells (**E**) and quantitative analysis (**F**) (*n* = 3/group). Bar graphs (**G**) showing the release levels of AST (U/L) and ALT (U/L) in the culture supernatant of AML12 cells (*n* = 3/group). ns *P* > 0.05; *P < 0.05; **P < 0.01; ***P < 0.001; ****P < 0.0001

To identify the functional receptor complex type of IL-24 in hepatocytes (IL20R1/IL20R2 vs. IL22R1/IL20R2), we first examined the activation of downstream STAT1 and STAT3. In both animal and cellular models, IL-24 significantly increased the p-STAT3/STAT3 ratio but did not markedly alter p-STAT1/STAT1 levels, preliminarily suggesting that IL-22R1/IL-20R2 may be its primary functional receptor (Fig. 7A-B, Fig. S4A). To validate this hypothesis, we knocked down IL20R1 and IL22R1 separately in AML12 cells (Table S2, Fig. S4B). Western blot results demonstrated (Fig. 7C-D, Fig. S4C) that compared to the PA group, IL20R1 knockdown increased p-STAT3 and p-AMPK expression while reducing LC3-II and p62 levels, effects similar to those observed in the PA + oeIL24 group. Conversely, IL22R1 knockdown reversed the protective effects of IL-24, with protein expression levels resembling those of the PA group. Notably, neither knockdown significantly affected STAT1 activation, further supporting that IL-24 primarily functions through IL22R1/IL20R2 rather than IL20R1/IL20R2. Phenotypically, Oil Red O staining (Fig. 7E-F) and biochemical assays of cell supernatants (Fig. 7G) were consistent with these molecular changes: under IL-24 intervention, IL22R1 knockdown exacerbated PA-induced lipid accumulation and cellular damage (elevated AST and ALT), whereas IL20R1 knockdown partially mimicked the protective effects of IL-24, alleviating lipid accumulation and hepatocyte injury. These results indicate that IL-24 regulates autophagy and mitigates hepatocyte steatosis and damage through the IL-22R1/IL-20R2 receptor complex.

### Multi-omics analysis reveals IL-24 ameliorates MASH-associated lipid deposition through metabolic reprogramming

**Fig. 8:**
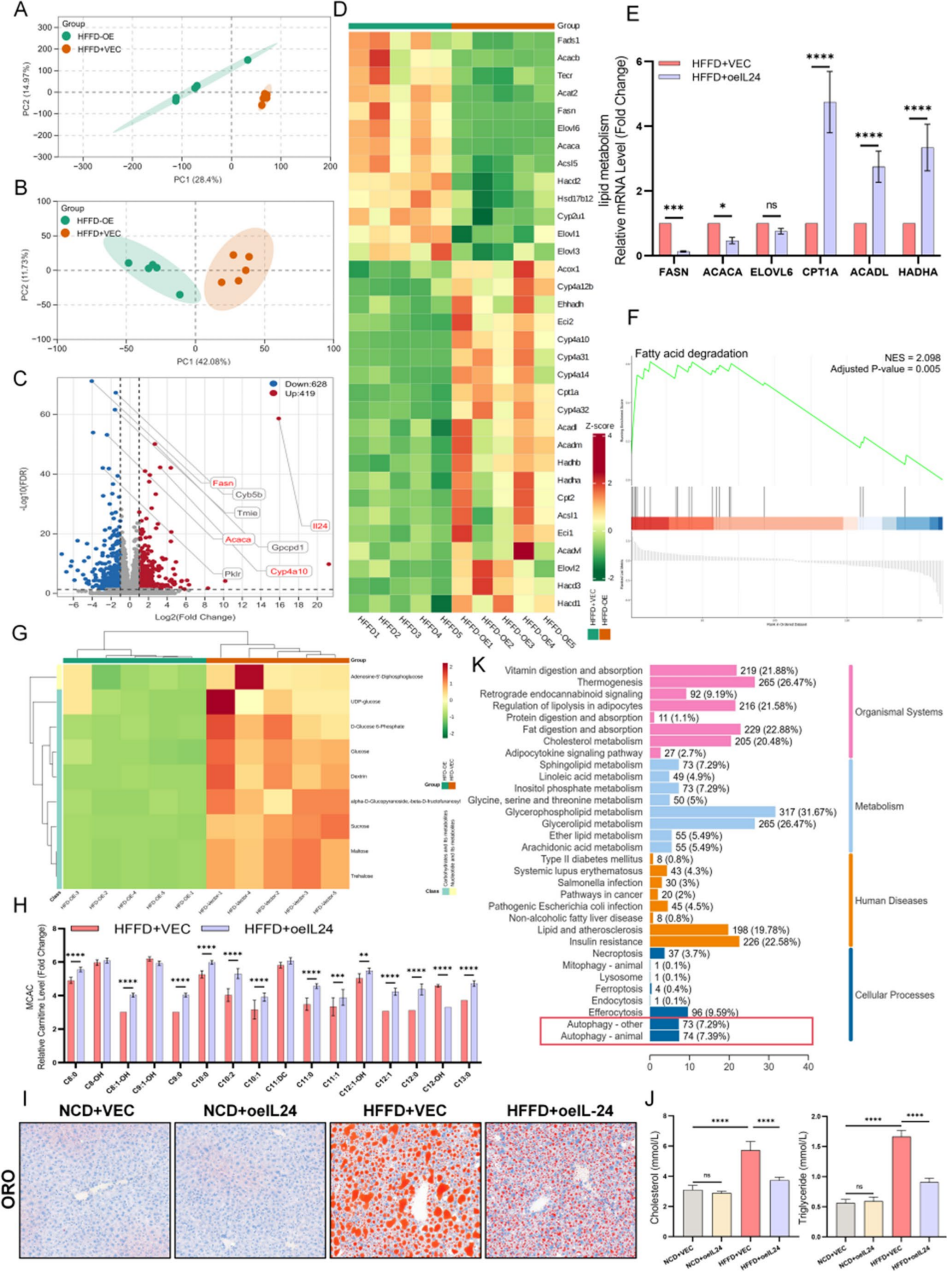
Multi-omics analysis of lipid metabolism and validation in vivo. (**A**) PCA of RNA-sequencing data shows distinct transcriptomic profiles between HFFD + VEC and HFFD + OE groups. (**B**) PCA of metabolomics data further distinguishes the two groups. (**C**, **D**) Volcano plot and hierarchical clustering heatmap analyses reveal differential expression patterns of lipid metabolism-related genes. (**E**) qPCR validation of key lipid metabolism-related mRNA expression in liver tissues (*n* = 6/group). (**F**) GSEA enrichment results showing lipid metabolism-related pathways in the RNA-seq dataset. (**G**) Hierarchical clustering heatmap of KEGG pathway-enriched metabolites demonstrates significant alterations in carbohydrate/nucleotide metabolism (n=/group). (**H**) Bar graph shows metabolic kinetics of medium-chain acylcarnitines (MCACs, C8-C14) in HFFD-induced mice with or without IL-24 overexpression (*n* = 5/group). (**I**) Representative photomicrographs of Oil Red O-stained liver sections. Quantification in Fig. S4E (*n* = 6/group). Scale bar: 200× magnification. (**J**) Bar graph shows serum cholesterol and triglyceride levels (mmol/L) in vivo (*n* = 6/group). (**K**) Bar graph shows the top 9 significantly enriched primary KEGG pathway categories of differential metabolites. ns *P* > 0.05; *P < 0.05; **P < 0.01; ***P < 0.001; ****P < 0.0001

The regulatory effects of IL-24 on lipid metabolism in MASH were investigated through integrated transcriptomic and metabolomic analyses. PCA revealed significant changes in gene expression (Fig. 8A) and metabolic profiles (Fig. 8B) following IL-24 treatment in HFFD-fed mice. Differential gene expression analysis demonstrated the downregulation of genes involved in lipid synthesis (FASN, ACACA) and the upregulation of genes associated with lipid catabolism (CPT1A, ACADL, HADHA) (qPCR-validated *P* < 0.05, Fig. 8C-E). GSEA further confirmed the significant enrichment of differentially expressed genes in fatty acid degradation pathways (NES = 2.098, Adjusted *P* = 0.005, Fig. 8F). Metabolomic profiling revealed a significant decrease in a range of carbohydrate-related metabolites (such as Adenosine-5’-Diphosphoglucose, UDP-glucose, D-Glucose 6-Phosphate, Glucose) upon IL-24 intervention (Fig. 8G), suggesting a decrease in glucose metabolic intermediates. Furthermore, we observed increased levels of short-, medium-, and long-chain acylcarnitines (CACs) (Fig. 8H, Fig. S4D). This finding, when integrated with the upregulation of fatty acid oxidation pathways in the transcriptomic data, is consistent with increased flux through β-oxidation in the liver. The concurrent occurrence of these glycolipid metabolic changes suggests that IL-24 intervention may promote metabolic reprogramming, a shift in metabolic priority from carbohydrate metabolism toward lipid metabolism, ultimately manifesting as reduced hepatic lipid droplets (ORO staining, Fig. 8I; quantification in Fig. S4E) and improved serum metabolic parameters (glucose, triglycerides, cholesterol, uric acid) (Fig. 8J, Fig. S4F). KEGG pathway enrichment analysis showed a significant clustering of differential metabolites within autophagy-related pathways (Fig. 8K). Further analysis of key metabolites (e.g., GDP, PE) indicated that their alteration patterns suggest potential enhancement of autophagic processes. In summary, these results demonstrate that IL-24 reprograms cellular metabolism toward catabolic processes, including autophagy, to maintain energy homeostasis.

## Discussion

Our study provides evidence supporting the regulatory role of IL-24 in MASH through a comprehensive approach integrating multiomics analyses, molecular biological assays, and pathological evaluations. We observed a significant reduction in IL-24 expression in MASLD patients and animal models, with levels inversely related to disease severity. In vivo, IL-24 intervention significantly improved HFFD-induced metabolic dysregulation and attenuated hepatic pathological damage, inflammation, and perisinusoidal fibrosis, thereby halting MASH progression. Mechanistic investigations suggest that IL-24 enhances autophagy-lysosome function via the AMPK/mTOR/TFEB axis, thereby promoting the clearance of intracellular lipids. Additionally, IL-24 preferentially signals through the IL-22R1/IL-20R2 receptor complex to activate STAT3 and also contributes to the activation of AMPK. Integrated multiomics analyses further support the notion of IL-24’s dual regulatory role in stimulating autophagy and reprogramming glucolipid metabolism by reducing carbohydrate metabolites while enhancing lipid catabolism. These findings indicate, for the first time, the protective role of IL-24 in MASH pathogenesis and, importantly, delineate its therapeutic mechanism involving the coordinated regulation of autophagy-lysosome function and metabolic reprogramming.

The IL-20 cytokine subfamily is an important member of the IL-10 family, playing key roles not only in maintaining epithelial homeostasis and integrity but also in regulating tissue repair processes following infection or inflammation [28]. As members of this family, IL-24 and IL-22 share the IL-22 receptor. The demonstrated hepatoprotective effects of IL-22 in liver diseases suggest that its homologous cytokine IL-24 also possesses therapeutic potential [29]. Although research beyond oncology remains limited, studies have demonstrated that in acute and chronic liver injury models, IL-24 protects hepatocytes, inhibits Ly6C + monocyte recruitment, and alleviates liver fibrosis by suppressing hepatic stellate cell activation and proliferation [17, 18]. Our research further reveals that in a MASH murine model, IL-24 intervention not only ameliorates hepatocyte injury and perisinusoidal fibrosis but also suppresses the infiltration of F4/80^+^ macrophages and Ly-6G^+^ neutrophils, thereby attenuating hepatic inflammation.

IL-24 exhibits complex molecular characteristics: its predicted monomeric molecular weight is 18.3 kDa, but mammalian cells produce various isoforms (18.3–35 kDa) through N-glycosylation at up to three sites [30]. These posttranslational modifications, along with potential oligomerization, contribute to its diverse biological activities and explain the difficulty in obtaining fully functional IL-24 from prokaryotic expression systems [31]. Furthermore, IL-24 exhibits low physiological stability, with excess free forms undergoing rapid degradation when not receptor-bound, a phenomenon associated with protective clearance [32]. Wang et al. [18] found that exogenously administered recombinant IL-24 failed to improve CCl4-induced liver injury, whereas endogenously expressed IL-24 conferred significant protection, suggesting functional limitations of eukaryotic-derived IL-24. Considering the low basal IL-24 expression detected in our study (CT values >30) and these challenges, we adopted an endogenous overexpression strategy to investigate the regulatory effects of IL-24 on MASH, ensuring the study of fully functional protein molecules.

Mechanistically, selective inhibition of IL20R1 and IL22R1 expression suggested that IL-24 preferentially activates STAT3 and AMPK through the IL-22R1/IL-20R2 receptor complex rather than IL-20R1/IL-20R2, consistent with previous studies [17]. This receptor specificity and its crosstalk with the AMPK signaling pathway provide a novel upstream mechanism to explain its metabolic and autophagic benefits. However, under physiological conditions, although IL-24 treatment effectively activates the IL22R1/IL20R2-STAT3 signaling pathway, it fails to alter the AMPK/mTOR/TFEB axis or the expression of autophagy-related proteins, which may be attributed to the following reasons: (1) Energy homeostasis constraints, whereby higher AMPK activation thresholds in basal states diminish pathway sensitivity; (2) A requirement for specific functional states, given that IL-24’s “bystander effects” are most evident under stress or pathological conditions [9–11]; and (3) Intrinsic negative feedback loops, through which endogenous mechanisms may counteract the function of exogenous IL-24 when basal autophagy is functioning normally. Notably, pathological states (e.g., AMPK inhibition or mTOR overactivation) markedly enhanced the system’s response to IL-24, potentially indicating compensatory pathways triggered by autophagy suppression. This key characteristic of state-dependent regulation—a functionality shared with IL-22 whereby potent effects are exerted in disease states with minimal homeostatic impact [33]—not only yields novel insights into the intricate control of IL-24’s biological functions but also highlights its therapeutic promise for metabolic disorders.

Hepatocytes exposed to a high-fat diet or toxic fatty acids (e.g., PA) exhibit significant impairment in autolysosome formation and lysosomal biogenesis, directly leading to defective lipid degradation and progression of MASLD [7]. With recent breakthroughs in nanotechnology, research has increasingly focused on exogenous intervention strategies, such as using acid-sensitive nanoparticles (acNPs) [34] or nanoscale silicate materials (Attapulgite) [35] to restore lysosomal acidification and thereby ameliorate autophagic dysfunction. In contrast, IL-24, as an endogenous cytokine, highlights unique advantages in endogenous signaling regulation. In this study, validation using the lysosomal inhibitor chloroquine (CQ) and the AMPK inhibitor compound C (CC) demonstrated that IL-24 exerts complex regulatory effects through the AMPK/mTOR/TFEB axis: this pathway not only restores autophagic flux but also concurrently mitigates inflammation and maintains metabolic homeostasis. This finding distinctly contrasts with previous studies predominantly focused on the mTOR pathway, thereby addressing gaps in earlier mechanistic research [15, 16]. Looking forward, IL-24 holds promise for combination with nanomaterials to enhance stability and targeting capability, enabling complementary applications of endogenous regulation and exogenous therapeutic strategies.

Furthermore, our findings suggest a potential mechanism by which IL-24 may regulate glycolipid metabolism through activation of the autophagy-lysosome pathway. In vivo experiments demonstrated that IL-24 intervention not only improved insulin resistance and glucose intolerance but also significantly reduced hepatic steatosis, accompanied by a restoration of serum metabolic parameters (i.e., blood glucose, lipids, uric acid). Metabolomics data support the notion of IL-24-mediated metabolic reprogramming from glucose catabolism to fatty acid oxidation. Decreased glucose intermediates may imply preferential ATP generation via β-oxidation, resulting in AMPK activation and mTOR inhibition [36]—concordant with our mechanistic studies. Network analysis revealed enrichment of differential metabolites within autophagy-related pathways, suggesting a possible functional link between IL-24-induced metabolic remodeling and autophagy activation. This synergistic interplay, mediated by the cellular energy sensing system (AMPK-mTOR-TFEB axis), facilitates the shift from anabolic to catabolic metabolic states, providing novel mechanistic insights into the regulation of energy homeostasis.

While this study provides valuable insights, several limitations should be acknowledged: First, it primarily focuses on the mechanistic role of IL-24 in regulating the AMPK/mTOR/TFEB signaling axis, while other potential mTOR-related pathways remain to be fully explored. Second, given prior evidence indicating limited therapeutic effects of exogenous IL-24 protein in liver diseases, this study adopted an endogenous overexpression strategy. Although this approach ensures protein activity, it cannot directly validate the therapeutic effects of purified IL-24 protein, which should be investigated in future studies. Furthermore, IL-24 protein inherently exhibits stability issues, and developing efficient targeted delivery systems or stabilizing protein structures (e.g., using nanomaterials) may help enhance its liver-specific therapeutic effects. Finally, due to ethical and technical constraints, validation of autophagic flux has currently been completed only in in vitro cellular models. Although histological and Western blot data from murine studies provide static correlative evidence, they are insufficient to directly establish dynamic causal relationships regarding autophagic flux alterations. To further clarify the clinical translational potential, subsequent studies require larger-scale animal models incorporating pathway inhibition or gene knockout experiments to dynamically monitor the real-time effects of IL-24 on autophagic flux in MASH models. Addressing these issues will contribute to a more comprehensive assessment of the potential clinical value of IL-24 in MASH therapy.

## Conclusion

This research provides perspectives on the molecular mechanisms by which IL-24 ameliorate MASH through modulation of the “autophagy-metabolism” network, thereby advancing our pathogenic understanding and the identification of therapeutic targets.These findings suggest innovative avenues for further research into precision therapy for MASH, with potential theoretical and clinical relevance.

## Supplementary Information

Below is the link to the electronic supplementary material.

Supplementary Methods (DOCX 25.1 KB)

Supplementary Tables and Figures (DOCX 3.64 MB)

Autophagy-related differential metabolites (XLSX 18.7 KB)

Supplementary Information (Liver HE and NAS Scoring) (PDF 1.83 MB)

## Data Availability

The data are available from the corresponding author upon reasonable request.
