# Supplementary material for: Intracellular IL-24 ameliorates lipid metabolic disorders in metabolic dysfunction-associated steatohepatitis by restoring the autophagy-lysosome pathway
Source: Cell Mol Life Sci. 2025 Nov 25;82(1):417. doi: 10.1007/s00018-025-05940-1 (PMC12647488; doi:10.1007/s00018-025-05940-1)
Supplement: Supplementary file 1 — Supplementary Methods (DOCX 25.1 KB) [file 18_2025_5940_MOESM1_ESM.docx]

**Supplementary Methods**

**Primary mouse hepatocytes isolation and Cell Culture**

Primary hepatocytes (PHs) were isolated from mouse livers using a standard two-step collagenase perfusion technique via the hepatic portal vein. Initially, livers were perfused with an EGTA-containing buffer (37°C, 5 mL/min flow rate, 8 min), followed by perfusion with collagenase I solution (Sigma-Aldrich; 0.5 mg/mL) at a flow rate of 2 mL/min for 10 min to ensure complete tissue digestion. The digested liver tissue was then gently dispersed in suspension buffer, and the resulting cell suspension was passed through a 70-µm cell strainer. PHs were subsequently purified by three rounds of low-speed centrifugation (50 ×g, 5 min, 4°C) to separate parenchymal hepatocytes from nonparenchymal cells. Cell viability, determined by trypan blue exclusion, was required to be greater than 85% for the hepatocytes to be used. These viable hepatocytes were seeded at a density of 1 × 10⁶ cells/mL in collagen-coated plates. The murine hepatocyte cell line Alpha Mouse Liver 12 (AML12) was obtained from Procell (CL-0602, Wuhan, China) and authenticated as being of murine origin through short tandem repeat profiling. Both PHs and AML12 cells were cultured in Dulbecco’s Modified Eagle Medium/Nutrient Mixture F-12 (DMEM/F12) (11320, Gibco) supplemented with 1× insulin-transferrin-selenium (ITS) (PB180429, Procell), 40 ng/mL dexamethasone (HY-14648, MCE), and 10% fetal bovine serum (FBS) (164210-50, Procell), with the addition of 1× penicillin-streptomycin (P/S) (PB180120, Procell) as needed. Cells were maintained at 37°C in a humidified atmosphere with 5% CO₂, and were tested quarterly for mycoplasma contamination.

**Construction and transfection of lentiviral vectors**

For lentiviral-mediated overexpression of IL-24, the full-length IL-24 (NM_053095) cDNA was PCR-amplified and subcloned into the pHBLV-CMV-MCS-3flag-EF1-puro vector. Lentiviral particles harboring the IL-24 expression cassette (LV-m-IL-24-3xflag-PURO) were produced by transfecting 293T cells. Control lentiviral particles, LV-Puro and LV-ZsGreen-PURO, were obtained from HanBio (Shanghai, China). Viral supernatants were used to infect AML12 and PHs. Stably transduced cells were selected by culture in the presence of 2 μg/mL puromycin for 7 days, whereas transient infections proceeded without any selection pressure.

**Cell transfection**

AML12 cells were seeded in six-well plates and incubated overnight. Cells were transiently transfected using Lipofectamine 3000 reagent (Invitrogen, Carlsbad, CA, USA). Small interfering RNAs (siRNAs) targeting IL20R1 and IL22R1 (designated as siIL20R1 and siIL22R1 respectively), along with a scrambled siRNA control, were synthesized by Gencefe (Beijing, China). The siRNA sequences are provided in Supplementary Table 2.

**Construction of the cellular model**

To create a palmitic acid (PA)-induced hepatocellular lipid accumulation model, 0.0307 g PA (HY-N0830, MCE) was dissolved in 3 mL of 0.1 M NaOH (221465, Sigma) at 75°C to generate sodium palmitate, which was subsequently mixed with 3 mL of 40% fat-free bovine serum albumin (BSA; CA1382-25g, COOLABER SCIENCE & TECHNOLOGY) solution. After vigorous vortexing, a 20 mM PA-20% BSA complex was formed (PA:BSA molar ratio = 6.6:1). The PA complex was diluted to required working concentrations using DMEM/F12 medium containing 1% FBS and co-incubated with hepatocytes for 24 hours. Control groups received equivalent volumes of 20% BSA solution diluted identically with DMEM/F12 medium containing 1% FBS to match final concentrations in experimental groups. To investigate underlying mechanisms, cells were treated for 24 hours with PA alone or in combination with Compound C (CC, 1 μM, HY-13418A, MCE) [1], rapamycin (RAPA, 0.25 μM, HY-10219, MCE)[2], or chloroquine (CQ, 10 μM, HY-17589A, MCE)[3].

**Histopathological examination**

Mouse liver specimens were fixed in 4% paraformaldehyde, embedded in paraffin, and sectioned into 4 µm-thick slices for histopathological analysis. For frozen sections used in Oil Red O (ORO) staining, tissues were embedded in OCT compound and cut into 8 µm sections, which were then stained following the manufacturer's protocol (G1260, Solarbio). For H&E staining, the sections were stained with hematoxylin for 10 min, eosin alcohol solution for 5 min, dehydrated, and overslipped. For Masson's Trichrome staining, the slides were immersed in Biebrich Scarlet-Acid Fuchsin solution for 10 min, stained with aniline blue for 10 s, dehydrated, and overslipped. For Sirius Red staining, the slides were incubated with Sirius Red staining solution for 30 min, dehydrated, and overslipped. Histopathological evaluation was performed using the NAFLD Activity Score (NAS) system, which quantitatively assesses steatosis (0-3), lobular inflammation (0-3), and hepatocyte ballooning (0-2), with total scores ranging 0-8 (scores ≥ 5 indicating MASH). To minimize observer bias, all histopathological assessments were independently performed by two blinded pathologists. Any discrepancies were resolved by a third senior hepatopathologist, whose evaluation served as the final adjudication. Images were captured using a light microscope and analyzed using ImageJ software for morphometric quantification.

**Quantitative RT-PCR**

Total RNA was isolated using TRIzol (15596026CN, Invitrogen) and 1 μg was reverse-transcribed into cDNA using the PrimeScript RT Reagent Kit (Takara, Dalian, China) based on the manufacturer's instructions. Quantitative real-time PCR (qRT-PCR) was performed using TB Green Premix Ex Taq II following the manufacturer's protocol. Gene expression was normalized to GAPDH as an internal control and quantified using the 2−ΔΔCt method. Primers are as follows: IL-24: 5′-AGAGCTGTTACCTTGCCCAC-3′ and 3′- GCTCTGCGGAACAGCAAAAA-5′; FASN: 5′-GGAGGTGGTGATAGCCGGTAT-3′ and 3′-TGGGTAATCCATAGAGCCCAG-5′; Acaca: 5′-ATGGGCGGAATGGTCTCTTTC-3′ and 3′-TGGGGACCTTGTCTTCATCAT-5′; Cpt1a: 5′-CTCCGCCTGAGCCATGAAG-3′ and 3′-CACCAGTGATGATGCCATTCT-5′; Acadl: 5′-TCTTTTCCTCGGAGCATGACA-3′ and 3′-GACCTCTCTACTCACTTCTCCAG-5′; Hadha: 5′-TGCATTTGCCGCAGCTTTAC-3′ and 3′-GTTGGCCCAGATTTCGTTCA-5′; Elovl6: 5′-GAAAAGCAGTTCAACGAGAACG-3′ and 3′-AGATGCCGACCACCAAAGATA-5′; IL20R1:5′-CAGCTTGAAGACCTACATCACCT-3′ and 3′-GGGTCCCAATCCACTACTGTTAT-5′; IL22R1:5′-AAGCCACCTGTACCTCCTAACTC-3′ and 3′-GACAGGGATCAGTACGTGTTCTT-5′; GAPDH: 5′-AGGTCGGTGTGAACGGATTTG-3′ and 3′-TGTAGACCATGTAGTTGAGGTCA-5′.

**Immunohistochemical staining**

Paraffin-embedded tissue sections were deparaffinized in xylene and rehydrated through graded ethanol series, followed by antigen retrieval in citrate buffer (pH 6.0) at 95°C for 20 minutes. After cooling to room temperature, endogenous peroxidase activity was blocked with 3% hydrogen peroxide for 15 minutes, and non-specific binding sites were blocked with 5% BSA for 30 minutes at room temperature. Sections were then incubated with primary antibodies against p62/SQSTM1 (1:200, PB0458, Boster), p-mTOR (Ser2448) (1:200, 67778-1-Ig, Proteintech), mTOR (1:400, 66888-1-Ig, Proteintech), F4/80 (1:500, 29414-1-AP, Proteintech), Ly-6G (1:800, AWA55855, Abiowell) and p-TFEB (Ser211) (1:200, AF3708, Affinity) overnight at 4°C. After washing, sections were incubated with HRP-conjugated secondary antibodies for 60 minutes at 37°C, followed by DAB (PV6000D, ZSGB-BIO) chromogen development and hematoxylin counterstaining.

**Immunofluorescence staining**

Cells were fixed with 4% paraformaldehyde for 15 min, followed by blocking with 5% BSA for 30 min at room temperature. The cells were incubated with primary antibodies, including anti-IL-24 (1:100, MAB2786, R&D Systems), anti-p62/SQSTM1 (1:200, PB0458, Boster), anti-p-mTOR (Ser2448) (1:200, 67778-1-Ig, Proteintech), and anti-TFEB (1:100, AWA43629, Abiowell), overnight at 4°C, followed by incubation with the following fluorescently labeled secondary antibodies for 60 min at 37°C: Dylight 488, Goat Anti-Rat IgG (1:200, A23240, Abbkine), Dylight 594, Goat Anti-Rabbit IgG (1:200, A23420, Abbkine), Dylight 488, Goat Anti-Rabbit IgG (1:200, A23220, Abbkine).

**LysoTracker Red and BODIPY 493/503 staining**

Cells were stained with 1:1000 BODIPY 493/503 (C2053S, Beyotime) for 15 min, 70 nM LysoTracker Red (C1046, Beyotime) for 60 min, and 1:100 Hoechst 33342 (C1027, Beyotime) for 10 min at 37°C to visualize lipid droplets, lysosomes, and nuclei, respectively. After incubation, cells were washed three times with phosphate-buffered saline (PBS) and immediately imaged under a fluorescence microscope.

**Autophagic flux analysis**

Cells were transduced with the adenovirus Ad-mCherry-EGFP-LC3 (HanBio, Shanghai, China) at an MOI of 40 for 24 hours to express the tandem fluorescent LC3 reporter. Autolysosomes (red fluorescent puncta) and autophagosomes (yellow-green fluorescent puncta) were then quantified using fluorescence microscopy to assess autophagic flux. Colocalization analysis of the fluorescent signals was performed using the ZEN software.

**Flow Cytometry**

To assess autophagic flux, both AML12 and PHs were analyzed using flow cytometry. Cells transfected with the Ad-mCherry-EGFP-LC3 adenovirus (HanBio, Shanghai, China) were harvested after the designated treatments for subsequent analysis. Fluorescence quantification was carried out using a CytoFLEX flow cytometer (Beckman Coulter, Brea, CA, USA). Compensation parameters were adjusted using control cells transduced with either Ad-mCherry or Ad-EGFP alone before conducting quantitative cell counts.

**Electron microscopy**

Transmission electron microscopy (TEM) was utilized for ultrastructural analysis, employing a Hitachi HT7800 microscope. Liver tissue samples were first fixed in 4% glutaraldehyde (Solarbio, P1127) and subsequently postfixed in 0.1M sodium cacodylate buffer. Dehydration was performed using a graded ethanol series, followed by embedding in epoxy resin. Ultrathin sections (70–90 nm) were stained with uranyl acetate and lead citrate. The Hitachi TEM system (HT7800) was used to analyze the morphology of autophagic structures, including autophagosomes and autolysosomes.

**Western blot**

Protein lysates were prepared from liver tissue or cells by lysis and homogenization in RIPA buffer supplemented with a proteinase inhibitor. Nuclear proteins were extracted using a Nuclear and Cytoplasmic Protein Extraction Kit (PK10014, Proteintech). The protein concentration was determined, and equal amounts of protein were separated by SDS-PAGE. Separated proteins were transferred to polyvinylidene difluoride (PVDF) membranes, which were then blocked with 5% fat-free FBS for 1 hour. Membranes were subsequently incubated overnight at 4°C with the following primary antibodies at the indicated dilutions: anti-Flag-Tag (1:1000, AB0030, Abways), anti-IL-24 (1:500, 26772-1-AP, Proteintech), anti-p-AMPK (Thr172) (1:500, AF3423, Affinity), anti-AMPK (1:500, AF6423, Affinity), anti-p-mTOR (Ser2448) (1:500, 67778-1-Ig, Proteintech), anti-mTOR (1:500, BM4182, Boster), anti-p-p70S6K (Thr389/Thr412) (1:500, AF3228, Affinity), anti-p70S6K (1:500, AF6226, Affinity), anti-p-TFEB (Ser211) (1:500, AF3708, Affinity), anti-LAMP2 (1:500, BM4357, Boster), anti-p62/SQSTM1 (1:1000, PB0458, Boster), anti-LC3 (1:500, 14600-1-AP, Proteintech), anti-STAT1 (1:500, AF6299, Affinity), anti-p-STAT1 (1:500, BM4541, Boster) , anti-STAT3 (1:800, 10253-2-AP, Proteintech), anti-p-STAT3 (1:500, 9134, CST), anti-α-SMA (1:500, BM3902, Boster), anti-Collagen I (1:1000, 14695-1-AP, Proteintech), anti-β-actin (1:5000, 81115-1-RR, Proteintech), anti-GAPDH (1:5000, A00227-1, Boster).After washing, membranes were incubated with a Dylight 800-conjugated Goat Anti-Rabbit IgG secondary antibody (1:5000, A23920, Abbkine) for 1 hour at 37°C. Protein band visualization and quantification were performed using the Odyssey fluorescence imaging system (LI-COR, USA) and ImageJ software, respectively.

**Transcriptomic analysis**

Total RNA was isolated from liver samples using the TRIzol method. Following extraction, the RNA was resuspended in 50 µL of DEPC-treated water. The concentration of the total RNA was determined using a Qubit fluorescence quantifier, and its integrity was assessed using a Qsep400 high-throughput biofragment analyzer. The resulting data were analyzed using Metware Cloud, a freely available online platform for data analysis (accessible at https://cloud.metware.cn).

**Metabolomics analysis**

Liver samples were retrieved from the -80 °C refrigerator and thawed on ice. Each sample was thoroughly homogenized under liquid nitrogen conditions and approximately 20 mg of the powdered tissue was accurately weighed. Subsequently, 1 mL of ice-cold extraction solvent (methyl tert-butyl ether (MTBE) : methanol (MeOH) = 3:1, v/v) containing an internal standard mixture was added. The mixture was vortexed continuously for 15 minutes, after which 200 μL of water was added. Following an additional 1 minute of vortexing, the sample was centrifuged at 12,000 × g for 10 minutes. A 200 μL aliquot of the upper organic layer was carefully collected and evaporated to dryness under a gentle stream of nitrogen or using a vacuum concentrator. The resulting dry extract was reconstituted in 200 μL of a solvent mixture (acetonitrile (ACN) : isopropanol (IPA) = 1:1, v/v) in preparation for LC-MS/MS analysis. Data analysis was performed using the Metware Cloud platform (https://cloud.metware.cn).

**Blood chemistry and cytokine quantification**

Serum IL-24 levels were measured using enzyme-linked immunosorbent assays specific for human (ZC-32427, ZCIBIO Technology Co., Ltd.) and mouse (ZC-37980, ZCIBIO Technology Co., Ltd.) sources, with absorbance read on a microplate reader (Rayto RT-6100, Rayto Life and Analytical Sciences, USA) following the respective manufacturers’ protocols. Mouse serum levels of alanine aminotransferase (ALT), aspartate aminotransferase (AST), glucose (GLU), total cholesterol, triglycerides (TG), and uric acid (UA) were quantified using a fully automated biochemical analyzer (Chemray 800, Rayto Life and Analytical Sciences, Shenzhen, China).

**REFERENCES**

1. Liu, Y. et al (2022) Salvia-Nelumbinis naturalis improves lipid metabolism of NAFLD by regulating the SIRT1/AMPK signaling pathway. BMC Complement Med Ther 22: 213. http://dai.org/10.1186/s12906-022-03697-9.

2. Chen, Y. et al (2020) Inositol-requiring enzyme 1α links palmitate-induced mTOR activation and lipotoxicity in hepatocytes. Am J Physiol Cell Physiol 319: C1130-c1140. http://dai.org/10.1152/ajpcell.00165.2020.

3. Sinha, R.A. et al (2014) Caffeine stimulates hepatic lipid metabolism by the autophagy-lysosomal pathway in mice. Hepatology 59: 1366-1380. <http://dai.org/10.1002/hep.26667.>
