# Supplementary material for: Intracellular IL-24 ameliorates lipid metabolic disorders in metabolic dysfunction-associated steatohepatitis by restoring the autophagy-lysosome pathway
Source: Cell Mol Life Sci. 2025 Nov 25;82(1):417. doi: 10.1007/s00018-025-05940-1 (PMC12647488; doi:10.1007/s00018-025-05940-1)
Supplement: Supplementary file 2 — Supplementary Tables and Figures (DOCX 3.64 MB) [file 18_2025_5940_MOESM2_ESM.docx]

**Supplementary Tables and Figures**

**Supplementary Table 1**

| Parameters | Healthy Control (n=150) | MASLD (n=150) | MASLD  with transaminitis (n=150) | Z / F / χ^2^ | P value |
| --- | --- | --- | --- | --- | --- |
| Age (years)^#^ | 39.0 (34.0-43.0) | 40.0 (35.0-47.3) | 39.0 (33.0-45.3) | 4.75 | 0.093 |
| Gender (F/M)^*^ | 48/102 | 85/65 | 129/21 | 96.00 | <0.001 |
| BMI (kg/m2)^#^ | 21.5 (20.2-23.1) | 26.1 (24.8-28.4) | 28.2 (25.7-30.8) | 232.75 | <0.001 |
| WBC (109/L)^#^ | 5.43 (4.82-6.17) | 6.66 (5.68-7.39) | 6.28 (5.48-7.21) | 53.92 | <0.001 |
| RBC (1012/L) | 4.91 ± 0.40 | 4.94 ± 0.39 | 4.88 ± 0.44 | 0.657 | 0.519 |
| Hb (g/L)^#^ | 147.1 (141.2-158.5) | 152.2 (141.0-159.0) | 153.1 (142.3-159.5) | 1.428 | 0.490 |
| PLT (109/L)^#^ | 241.8 (210.1-268.6) | 253.4 (214.0-292.0) | 244.1 (210.6-277.1) | 4.09 | 0.129 |
| AST (U/L)^#^ | 17.0 (15.0-20.0) | 19.0 (17.0-22.0) | 32.5 (27.0-43.3) | 260.83 | <0.001 |
| ALT (U/L)^#^ | 15.0 (11.0-20.3) | 25.0 (19.0-33.0) | 56.0 (48.0-71.3) | 317.32 | <0.001 |
| ALP (U/L)^#^ | 65.0 (51.0-76.0) | 65.0 (51.0-81.0) | 73.0 (68.0-77.0) | 32.04 | <0.001 |
| GGT (U/L)^#^ | 22.0 (15.0-26.0) | 36.5 (26.0-54.0) | 36.0 (27.0-54.0) | 158.09 | <0.001 |
| ALB(g/L) | 47.4 ± 3.2 | 47.8 ± 2.3 | 47.5 ± 2.6 | 0.30 | 0.395 |
| Glu (mmol/L)^#^ | 5.18 (4.89-5.53) | 5.49 (5.15-6.36) | 5.63 (5.19-6.05) | 47.08 | <0.001 |
| TC (mmol/L)^#^ | 4.48 (4.05-4.95) | 5.05 (4.46-5.59) | 5.17 (4.68-5.99) | 70.66 | <0.001 |
| TG (mmol/L)^#^ | 0.93 (0.72-1.23) | 1.81 (1.31-2.81) | 1.88 (1.34-2.54) | 157.79 | <0.001 |
| HDL-C (mmol/L)^#^ | 1.41 (1.22-1.55) | 1.15 (1.02-1.30) | 1.19 (1.06-1.35) | 75.95 | <0.001 |
| LDL-C (mmol/L)^#^ | 2.53 (2.26-2.81) | 2.97 (2.56-3.37) | 3.08 (2.75-3.69) | 88.25 | <0.001 |
| CAP (dB/m)^#^ | 216.55 (209.18-221.35) | 289.80 (275.75-301.13) | 303.25 (289.23-326.55) | 307.67 | <0.001 |
| LSM (kPa)^#^ | 5.10 (4.38-5.80) | 5.75 (4.90-6.70) | 6.30 (5.60-7.50) | 93.22 | <0.001 |
| IL-24 (pg/ml)^#^ | 220.545 (205.08–245.83) | 191.25 (171.97–212.61) | 116.02 (95.48–135.75) | 337.71 | <0.001 |

**Table S1:** **Analysis of serum IL24 levels and other clinical characteristics in MASLD patients and healthy controls.** BMI, body mass index; WBC, white blood cells; RBC, red blood cells; Hb, hemoglobin; PLT, platelets; AST, aspartate aminotransferase; ALT, alanine aminotransferase; ALP, alkaline phosphatase; GGT, gamma-glutamyl transferase; ALB, albumin; Glu, glucose; TC, total cholesterol; TG, triglycerides; HDL-C, high-density lipoprotein cholesterol; LDL-C, low-density lipoprotein cholesterol; CAP, controlled attenuation parameter; LSM, liver stiffness measurement; IL-24, interleukin-24. Values are expressed as mean ± SD or median (IQR). * means Fisher’s precision probability test. # means Kruskal-Wallis H test.

**Supplementary Table 2**

| Target Gene | Designation | Sequence (5′ to 3′) | Choose |
| --- | --- | --- | --- |
| IL20R1 | siIL20R1-1 | Sense Strand: GCUACUUGGUUUACCGUUATT  Antisense Strand：UAACGGUAAACCAAGUAGCTT | √ |
|  | siIL20R1-2 | Sense Strand: GAGUCUACACGGAGUUGAATT  Antisense Strand：UUCAACUCCGUGUAGACUCTT |  |
|  | siIL20R1-3 | Sense Strand: CCAGAGGAAGAGACAUCUATT  Antisense Strand：UAGAUGUCUCUUCCUCUGGTT |  |
| IL22R1 | siIL22R1-1 | Sense Strand: GGACCUUUCUCUACAGAAATT  Antisense Strand：UUUCUGUAGAGAAAGGUCCTT |  |
|  | siIL22R1-2 | Sense Strand: CCGAUAUUGUCCAAGGAAATT  Antisense Strand：UUUCCUUGGACAAUAUCGGTT | √ |
|  | siIL22R1-3 | Sense Strand: CCGUCUACAGUGUGGAAUATT  Antisense Strand：UAUUCCACACUGUAGACGGTT |  |
| Negative control | siNC | Sense Strand: UUCUCCGAACGUGUCACGUTT  Antisense Strand：ACGUGACACGUUCGGAGAATT | √ |

**Table S2: List of siRNA sequences targeting IL20R1 and IL22R1.**

**Supplementary Figure 1**


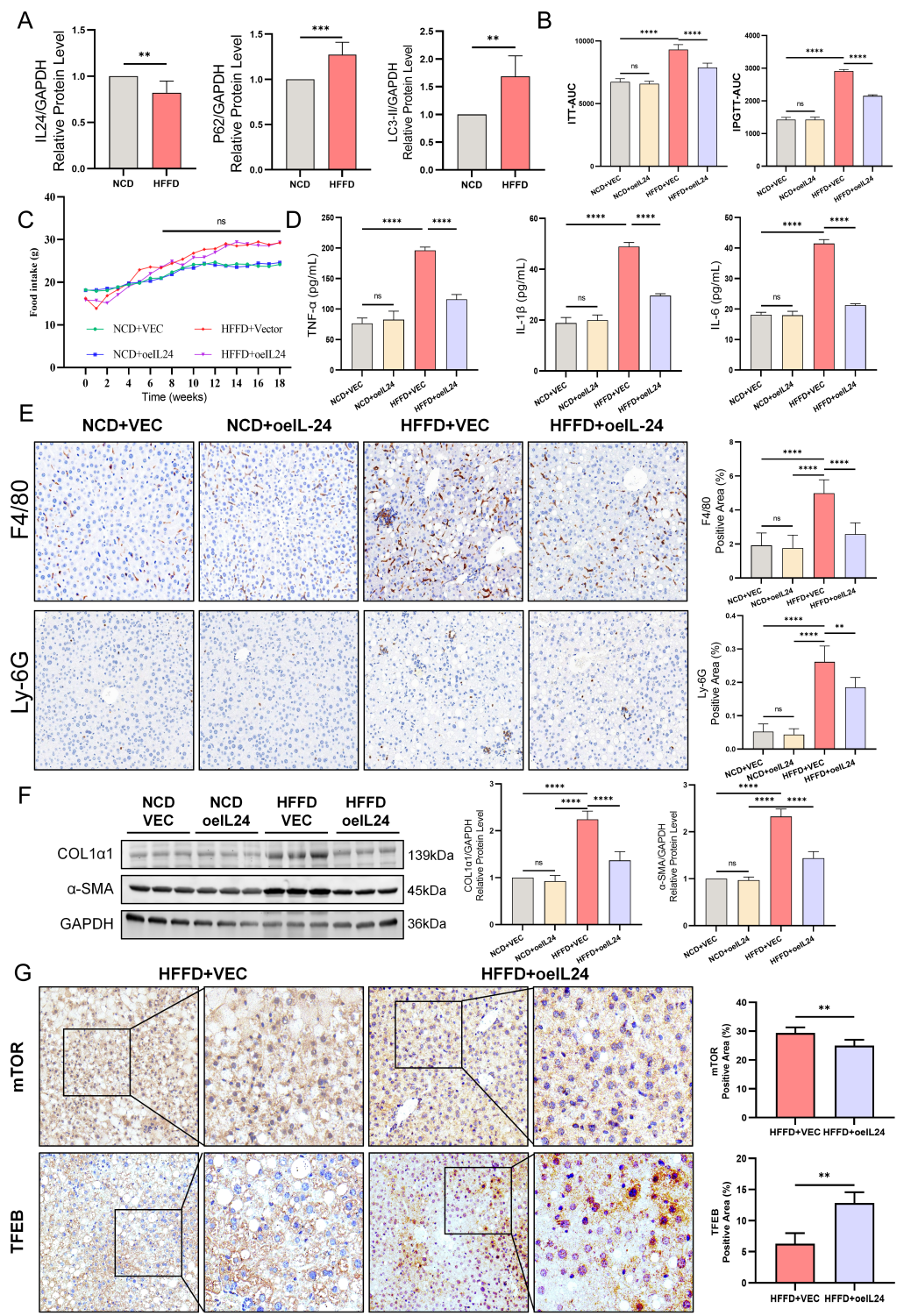


**Figure S1:** **In vivo validation of IL-24's effects on metabolism, inflammation, fibrosis, and autophagy-related signaling in MASH.** (A) Quantification of protein levels for IL-24, LC3-II, and p62 in liver tissues, corresponding to Figure 1H. (B) Quantification of IPGTT and ITT results. Bar graphs show the Area Under the Curve (AUC) over 120 minutes for IPGTT and ITT (n=6/group). Corresponding to Figure 1E-F. (C) Weekly average food intake per mouse in different groups over the 18-week feeding period. Mice were fed with a normal chow diet (NCD) or a high-fat/high-fructose diet (HFFD) and treated with or without IL-24 as indicated (n=3 cages per group; 2 mice per cage). No significant difference in food intake between HFFD+VEC and HFFD+oeIL-24 groups. (D) Serum levels of pro-inflammatory cytokines (TNF-α, IL-6, and IL-1β) in mice (n=6/group). (E) Immunohistochemical staining of F4/80 and Ly-6G in representative liver tissues from the HFFD mouse model (n = 6/group). Scale bars are shown for 200×magnification. (F) Western blot analysis of fibrotic markers (α-SMA and COL1α1) in liver tissues (n = 6/group). (G) Immunohistochemical staining of mTOR and TFEB in representative liver tissues from the HFFD mouse model (n = 6/group). Scale bars are shown for 200×and 400×magnification. ns P > 0.05; *P < 0.05; **P < 0.01; ***P < 0.001; ****P < 0.0001. All data are illustrated as mean ± SD.

**Supplementary Figure 2**


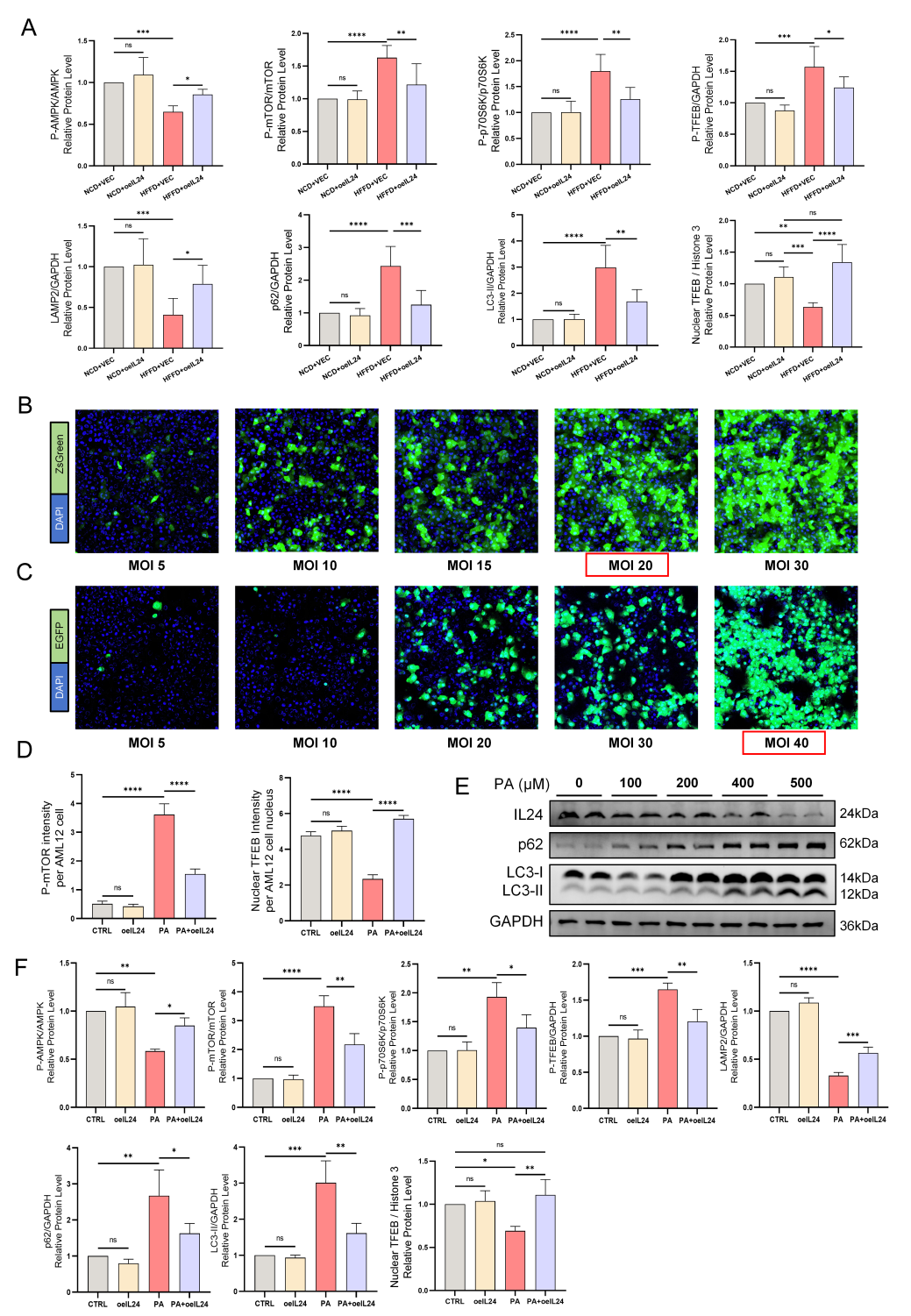


**Figure S2: In vitro and in vivo modulation of the AMPK/mTOR/TFEB pathway and optimization of viral transduction conditions.** (A) Densitometry analysis of Western blot for proteins in the AMPK/mTOR pathway (i.e., p-AMPK/AMPK, p-mTOR/mTOR, p-p70S6K/p70S6K, p-TFEB, TFEB, LAMP2, p62, and LC3-II) from liver lysates, corresponding to Figure 3D. (B) Lentiviral MOI titration. MOI optimization for LV-ZsGreen-PURO lentivirus. MOI values (5, 10, 15, 20, 30) were tested to achieve 70-80% transduction efficiency (ZsGreen+ cells) with >80% viability. Scale bars: 200×magnification. (C) Adenoviral MOI titration. MOI optimization for Ad-EGFP. MOI values (5, 10, 20, 30, 40) were tested to achieve 70-80% transduction efficiency (EGFP+ cells) with >80% viability. Scale bars: 200×magnification. (D) Quantification of immunofluorescence staining for p-mTOR phosphorylation and TFEB nuclear translocation in AML12 cells, corresponding to Figure 4B. (n = 3/group). (E) Western blot of LC3II and p62 expression in PHs treated with increasing PA concentrations for 24 hours. (F) Quantification of protein levels for P-AMPK/AMPK, P-mTOR/mTOR, P-p70S6K/p70S6K, P-TFEB, TFEB, LAMP2, p62, and LC3-II *in vitro*, corresponding to Figure 4E. ns P > 0.05; *P < 0.05; **P < 0.01; ***P < 0.001; ****P < 0.0001. All data are illustrated as mean ± SD.

**Supplementary Figure 3**


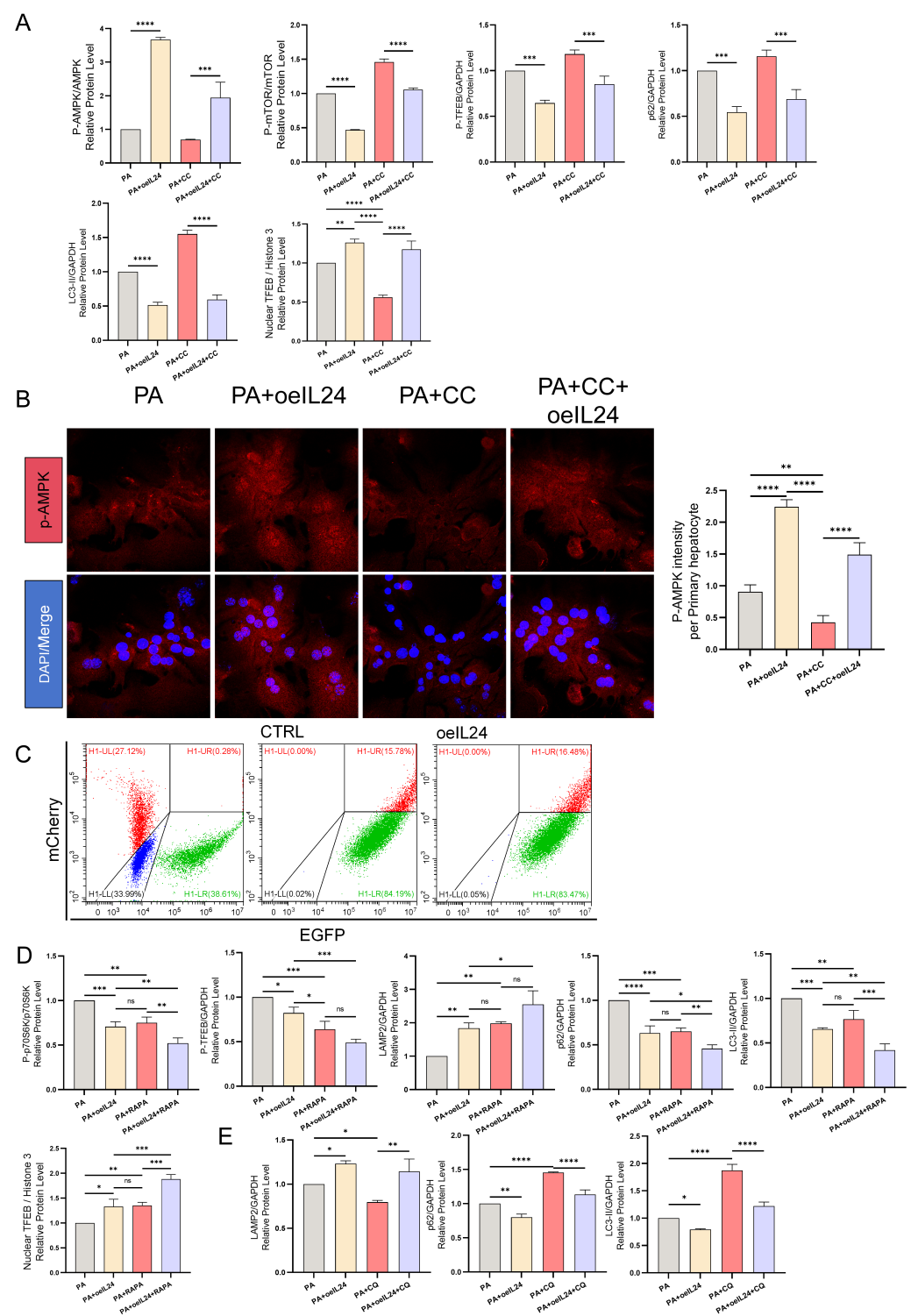


**Figure S3:** **IL-24 enhances autophagic flux and lysosomal function through AMPK/mTOR/TFEB signaling.** (A) Quantification of protein levels for P-AMPK/AMPK, P-mTOR/mTOR, P-TFEB, TFEB, p62, and LC3-II *in vitro*, corresponding to Figure 4F. (B) Representative immunofluorescence images show p-AMPK (red) with DAPI (blue) nuclear counterstaining in PHs. Scale bars: 200×magnification. (C) Autophagic flux in PHs transduced with Ad-mCherry-EGFP-LC3 relative to CTRL and oeIL-24 groups, corresponding to Figure 5C. (D) Quantification of protein levels for p-p70S6K/p70S6K, p-TFEB, TFEB, LAMP2, p62, and LC3-II in vitro, corresponding to Figure 5D. (E) Quantification of protein levels for LAMP2, p62, and LC3-II in vitro, corresponding to Figure 5E. (n = 3/group). ns P > 0.05; *P < 0.05; **P < 0.01; ***P < 0.001; ****P < 0.0001. All data are illustrated as mean ± SD.

**Supplementary Figure 4**


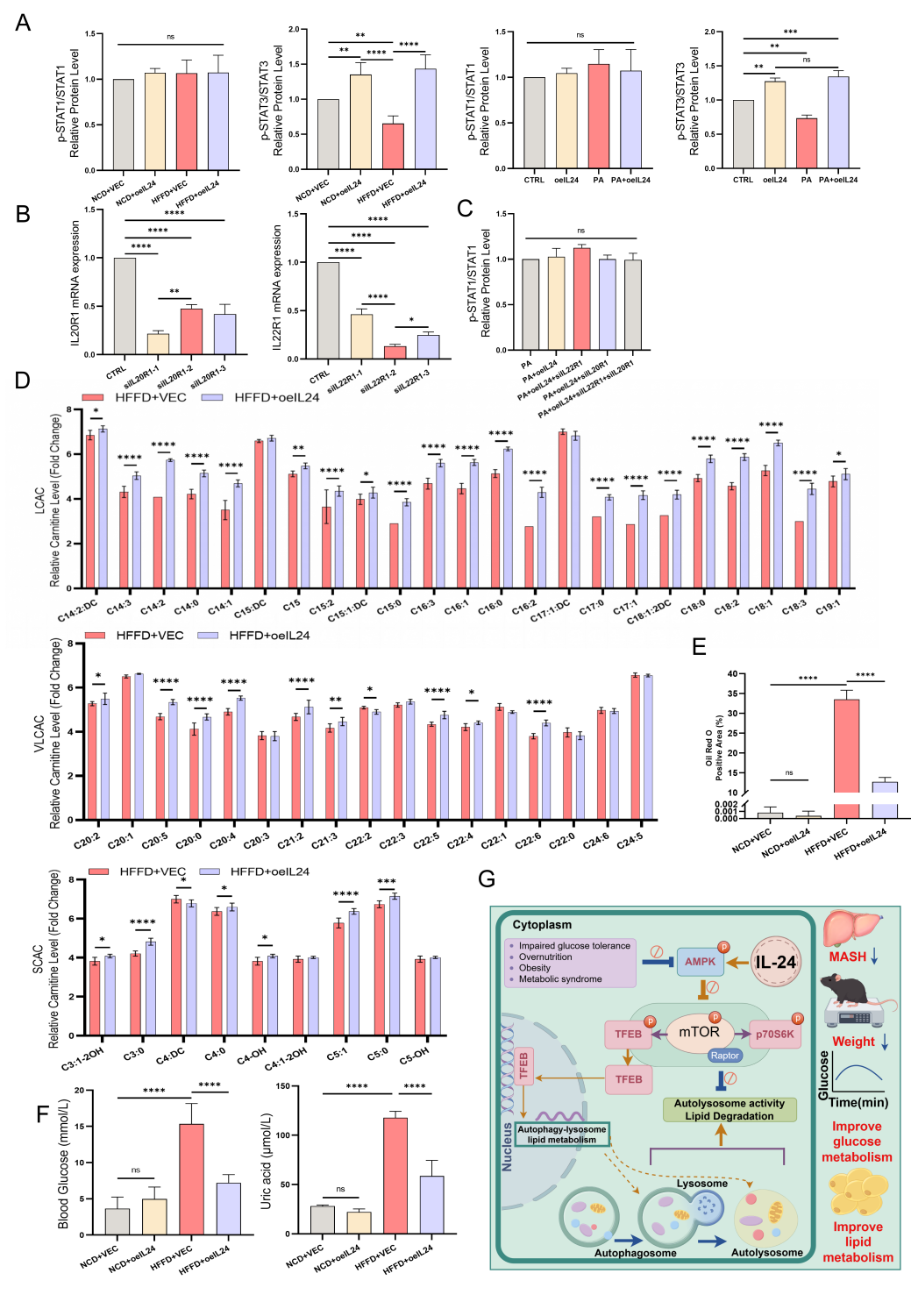


**Figure S4:** **IL-24 receptor signaling, metabolic profiling, and systemic improvements in MASH mice.** (A) Quantification of protein levels for p-STAT1/STAT1 and p-STAT3/STAT3 in *vitro* (n = 6/group) and in *vitro* (n = 3/group), corresponding to Figure 7A-B. (B) Screening of siRNA sequences for efficient knockdown of IL20R1 and IL22R1 in AML12 cells (n=3). (C) Quantification of protein levels for p-STAT1/STAT1 in *vitro* (n = 3/group), corresponding to Figure 7C. (D) Bar graphs show the metabolic profiles of acylcarnitines in HFFD-induced mice with or without IL-24 overexpression, including short-chain (SCACs, C2-C6), long-chain (LCACs, C14-C19), and very-long-chain (VLCACs, ≥C20) acylcarnitines (n = 5/group), corresponding to Figure 8H. (E) Quantification of Oil Red O staining confirms reduced hepatic lipid content, corresponding to Figure 8I. (F) Analysis of blood glucose (mmol/L) and serum UA (μmol/L) levels between groups (n = 6/group). (G) Graphical abstract designed by Figdraw.com. ns P > 0.05; *P < 0.05; **P < 0.01; ***P < 0.001; ****P < 0.0001. All data are illustrated as mean ± SD.
