# Supplementary material for: Intracellular IL-24 ameliorates lipid metabolic disorders in metabolic dysfunction-associated steatohepatitis by restoring the autophagy-lysosome pathway
Source: Cell Mol Life Sci. 2025 Nov 25;82(1):417. doi: 10.1007/s00018-025-05940-1 (PMC12647488; doi:10.1007/s00018-025-05940-1)
Supplement: Supplementary file 4 — Supplementary Information (Liver HE and NAS Scoring) (PDF 1.83 MB) [file 18_2025_5940_MOESM4_ESM.pdf]

HFFD Group

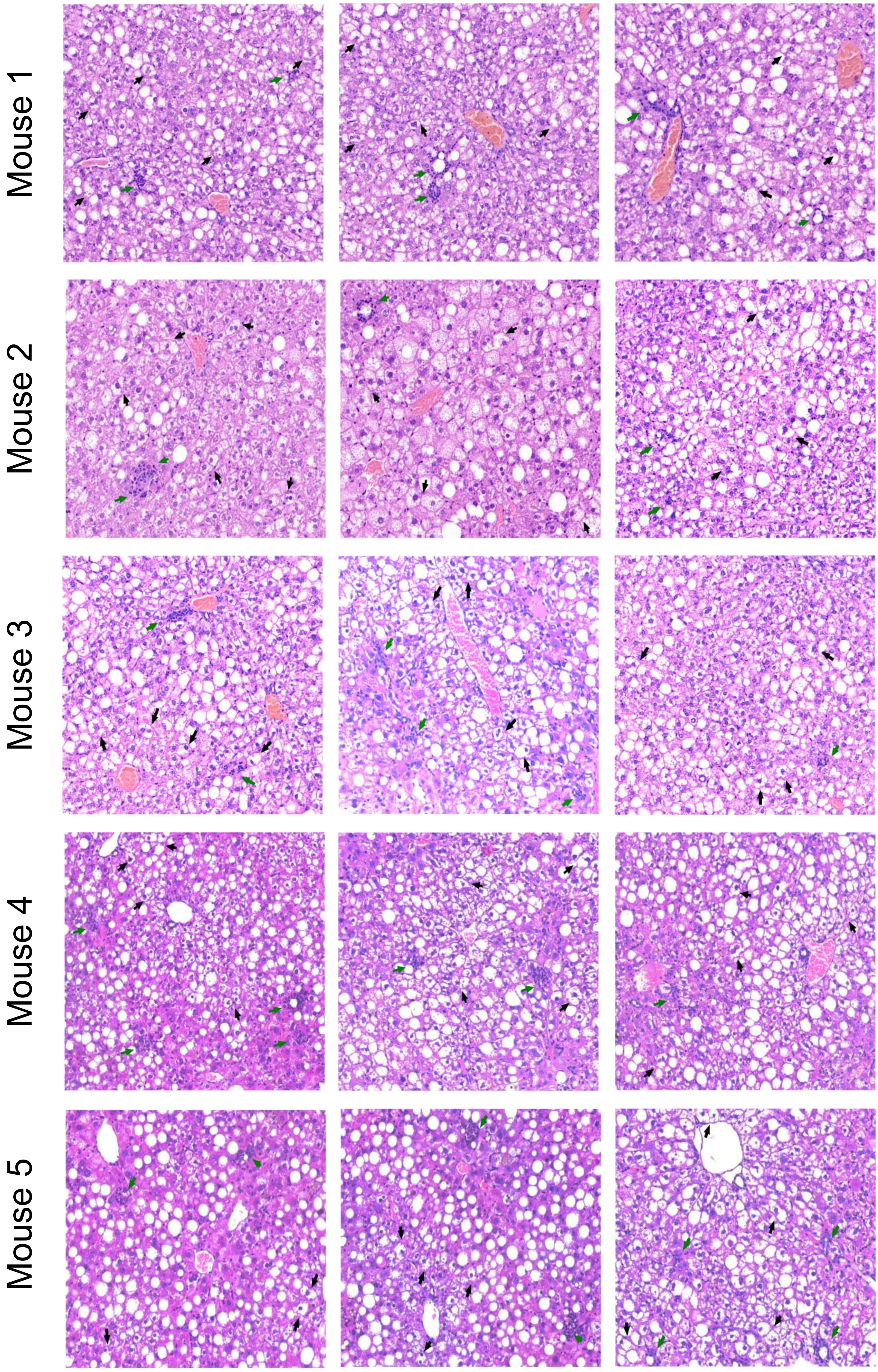

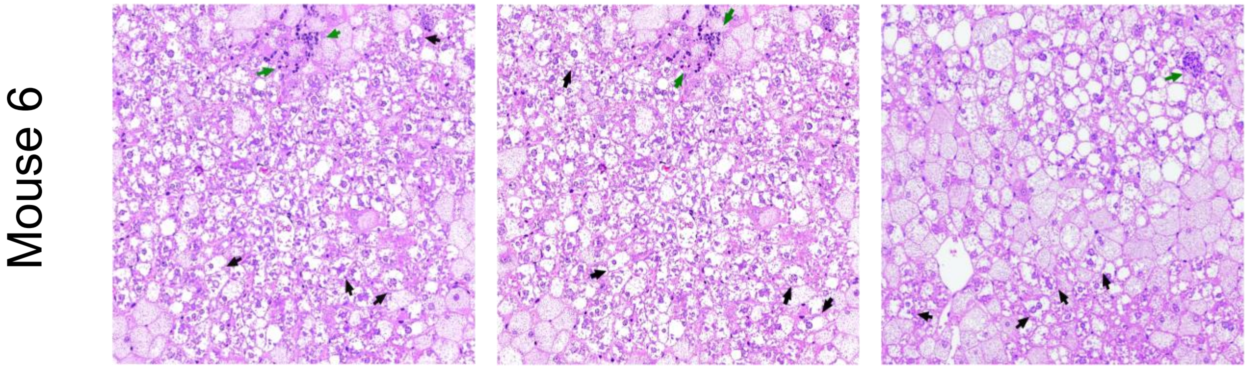

HFFD+oeIL24 Group

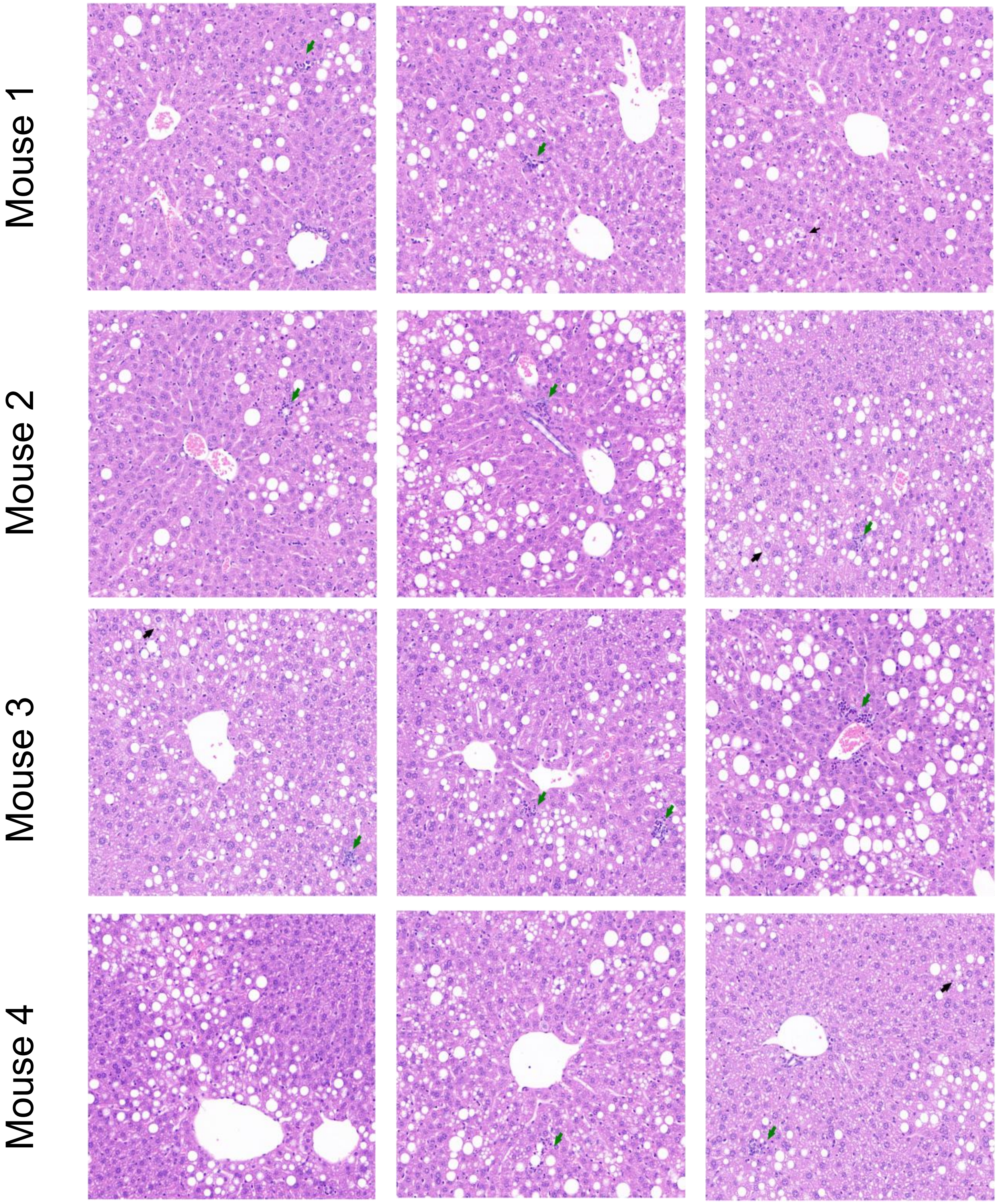

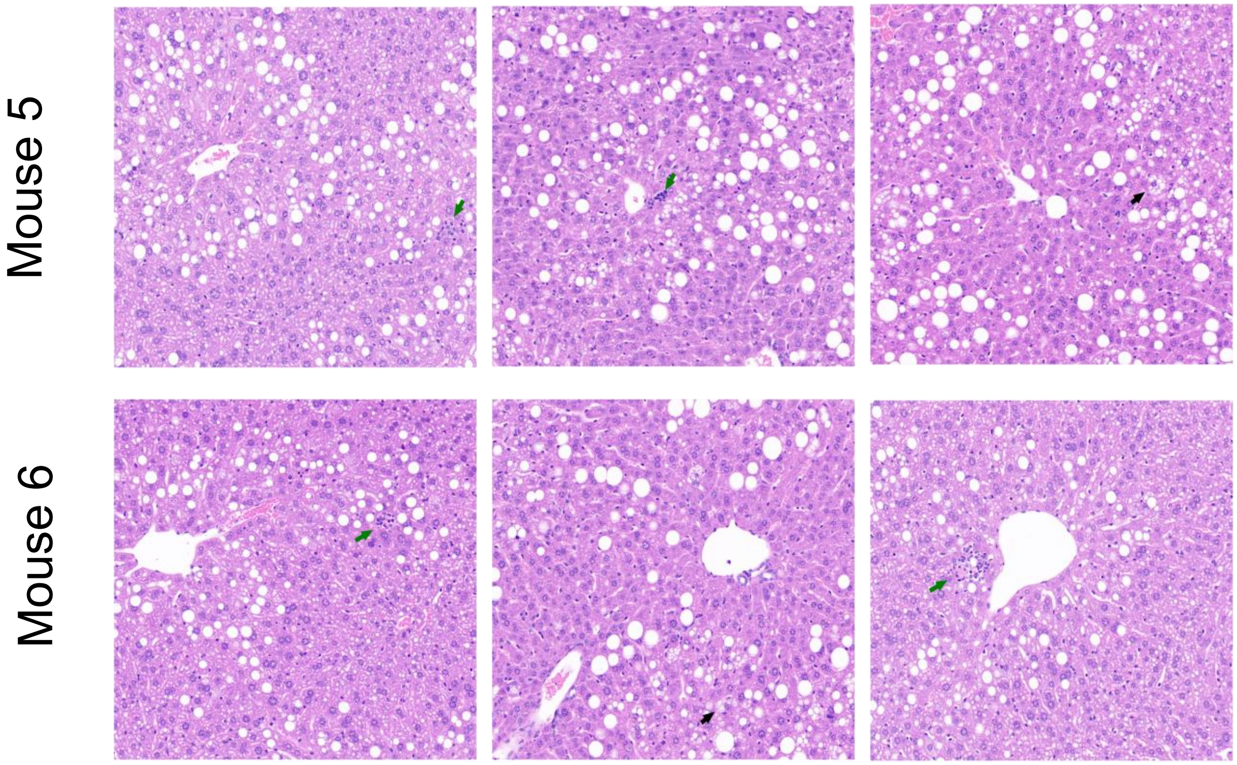

Figure 1: Representative H&E-stained liver sections used for NAFLD Activity Score (NAS) assessment. Green arrows indicate inflammatory foci; black arrows denote hepatocyte ballooning degeneration. Scale bar: 200× magnification.

Group	Sample Number	Steatosis			Lobular Inflammation			Hepatocyte Ballooning			Total score
		review 1	review 2	Final Score	review 1	review 2	Final Score	review 1	review 2	Final Score	
CTRL	Mouse 1	0	0	0	0	0	0	0	0	0	**0**
	Mouse 2	0	0	0	0	0	0	0	0	0	**0**
	Mouse 3	0	0	0	0	0	0	0	0	0	**0**
	Mouse 4	0	0	0	0	0	0	0	0	0	**0**
	Mouse 5	0	0	0	0	0	0	0	0	0	**0**
	Mouse 6	0	0	0	0	0	0	0	0	0	**0**
oeIL24	Mouse 1	0	0	0	0	0	0	0	0	0	**0**
	Mouse 2	0	0	0	0	0	0	0	0	0	**0**
	Mouse 3	0	0	0	0	0	0	0	0	0	**0**
	Mouse 4	0	0	0	0	0	0	0	0	0	**0**
	Mouse 5	0	0	0	0	0	0	0	0	0	**0**
	Mouse 6	0	0	0	0	0	0	0	0	0	**0**
HFFD+Vec	Mouse 1	3	3	3	2	2	2	2	2	2	**7**
	Mouse 2	3	3	3	1	2	1	2	2	2	**6**
	Mouse 3	3	3	3	2	1	1	1	2	2	**6**
	Mouse 4	3	3	3	3	2	2	2	2	2	**7**
	Mouse 5	3	3	3	2	2	2	2	2	2	**7**
	Mouse 6	3	3	3	2	1	1	2	2	2	**6**
HFFD+oeIL24	Mouse 1	1	1	1	1	1	1	1	1	1	**3**
	Mouse 2	1	2	2	1	1	1	0	1	1	**4**
	Mouse 3	2	2	2	1	2	2	0	1	0	**4**
	Mouse 4	2	2	2	1	1	1	1	1	1	**4**
	Mouse 5	2	2	2	1	1	1	1	1	1	**4**
	Mouse 6	1	2	2	1	1	1	1	1	1	**4**

Table 1: NAFLD Activity Score and Overall Assessment
